# Poly(ADP-ribose) polymerase-1 antagonizes DNA resection at double-strand breaks

**DOI:** 10.1038/s41467-019-10741-9

**Published:** 2019-07-04

**Authors:** Marie-Christine Caron, Ajit K. Sharma, Julia O’Sullivan, Logan R. Myler, Maria Tedim Ferreira, Amélie Rodrigue, Yan Coulombe, Chantal Ethier, Jean-Philippe Gagné, Marie-France Langelier, John M. Pascal, Ilya J. Finkelstein, Michael J. Hendzel, Guy G. Poirier, Jean-Yves Masson

**Affiliations:** 1 Genome Stability Laboratory, CHU de Québec Research Center, HDQ Pavilion, Oncology Division, 9 McMahon, Québec City, QC G1R 3S3 Canada; 2 Department of Molecular Biology, Medical Biochemistry and Pathology, Laval University Cancer Research Center, Québec City, QC G1V 0A6 Canada; 3 Department of Oncology, Faculty of Medicine and Dentistry, University of Alberta, 11560 University Avenue, Edmonton, AL T6G 1Z2 Canada; 4 Department of Molecular Biosciences, University of Texas at Austin, Austin, TX 78712 USA; 5 CHU de Québec Research Center, CHUL Pavilion, Oncology Division, 2705 Boulevard Laurier, Québec City, QC G1V 4G2 Canada; 6 Biochemistry and Molecular Medicine, Université de Montréal, 2900 Boulevard Edouard-Montpetit, Pavillon Roger-Gaudry, Montréal, QC H3T 1J4 Canada

**Keywords:** Biochemistry, DNA, Cancer, Cell biology, Cell signalling

## Abstract

PARP-1 is rapidly recruited and activated by DNA double-strand breaks (DSBs). Upon activation, PARP-1 synthesizes a structurally complex polymer composed of ADP-ribose units that facilitates local chromatin relaxation and the recruitment of DNA repair factors. Here, we identify a function for PARP-1 in DNA DSB resection. Remarkably, inhibition of PARP-1 leads to hyperresected DNA DSBs. We show that loss of PARP-1 and hyperresection are associated with loss of Ku, 53BP1 and RIF1 resection inhibitors from the break site. DNA curtains analysis show that EXO1-mediated resection is blocked by PARP-1. Furthermore, PARP-1 abrogation leads to increased DNA resection tracks and an increase of homologous recombination in cellulo. Our results, therefore, place PARP-1 activation as a critical early event for DNA DSB repair activation and regulation of resection. Hence, our work has direct implications for the clinical use and effectiveness of PARP inhibition, which is prescribed for the treatment of various malignancies.

## Abstract (web summary)

Poly(ADP-ribose) polymerase-1 (PARP-1) facilitates local chromatin relaxation and the recruitment of DNA repair factors at double strand breaks site (DSBs). Here the authors reveal that PARP-1 acts as a critical regulator of DNA end resection of DSBs.

## Introduction

Each day, the eukaryotic genome is confronted with up to 50 endogenous DNA double-strand breaks (DSBs)^1^. These are extremely hazardous for a cell, because if left unrepaired, DSBs can have pathological consequences, such as cell death, or drive cells to genomic instability and tumor development. The cellular response to DNA damage involves an intricate network of enzymes responsible for sensing, signaling, and repairing damaged DNA, as well as the regulation of cell cycle checkpoints that collectively maintain genomic integrity^2^.

Poly(ADP-ribose) polymerase-1 (PARP-1) is an abundant and ubiquitous nuclear protein that uses NAD^+^ to synthesize a multibranched polyanion composed of ADP-ribose moieties, giving rise to poly(ADP-ribose) (PAR), onto itself or a variety of target proteins. Protein ADP-ribosylation permits the transfer of the ADP-ribose moiety from NAD^+^ to the side chain of several amino acids^3–5^. Predominant biological processes targeted by PARylation include RNA splicing, processing, and maturation, DNA replication, and transcription as well as the DNA damage response (DDR)^3,6,7^. PARP-1 acts as a highly sensitive sensor for DNA damage and rapidly produces PAR at newly generated DNA DSBs. This promotes local chromatin relaxation due to its negative charge^8^ and histone displacement^9^, as well as facilitating the recruitment of repair factors, such as MRE11^10^. Several PAR-binding modules orchestrate the relocation of DDR-associated factors in addition to the accumulation of intrinsically disordered proteins through an intracellular liquid demixing mechanism^11,12^. PARP-1 is responsible for 80–90% of the global PAR synthesis following DNA strand breakage^13^. The dynamic turnover of PAR within seconds to minutes is executed by poly(ADP-ribose) glycohydrolase, the main PAR-degrading enzyme, which possesses both endoglycosidic and exoglycosidic activities, thereby enabling a new round of DNA damage signaling^14^. More recently, it has been shown that PARP-1 inhibition increases the speed of fork elongation and does not cause fork stalling, which is in contrast to the accepted model in which inhibitors of PARP induce fork stalling and collapse^15^. It was also recently shown that PARP-1 is a sensor of unligated Okazaki fragments during DNA replication^16^ and cells deficient in ribonucleotide excision repair are sensitized to PARP inhibition^17^.

PARP-1 is the best-characterized member of the diphtheria toxin-like ADP-ribosyl transferases (ARTDs) family of proteins. Among the 17 ARTDs members, only PARP-1, PARP-2, and PARP-3 are activated by DNA strand breaks^18–20^. De Murcia and colleagues provided the first evidence implicating PARP-1 in DNA repair by demonstrating that PARP-1-deficient mice are highly sensitive to γ-irradiation^21^. PARP-1 plays a critical role in DSB sensing and we have shown that PARP-1 recruitment and activation occur within 100 ms after introduction of DSBs. This makes PARP-1 activation one of the earliest and most critical events in the sensing of DSBs. Consistent with this, PARP-1 activity increases the rate of recruitment of the MRE11-RAD50-NBS1 (MRN) complex^10^ and stimulates Ku binding in Dictyostelium^22^. Structural analyses of PARP-1 have shown that PARP-1 binds DSBs. It does so through interactions with its zinc fingers and a WGR domain^23–25^.

In mammalian cells, most DSBs are repaired using long homologous sequences (homologous recombination (HR)), microhomology-mediated end joining, or no homology end joining (NHEJ). A key event that controls the DSB repair pathway choice is DNA end resection, which is characterized by 5′ to 3′ degradation of one strand at each side of the break. HR is initiated by CtBP-interacting protein (CtIP), a key molecular switch that controls DNA end resection and interacts with BRCA1. Although BRCA1 is a CtIP-interacting protein, there are conflicting reports on the roles of BRCA1 in DNA resection. While an early report found that disrupting the CtIP–BRCA1 interaction in DT40 cells diminished DNA-end resection^26^, a later report showed that CtIP mutated at Ser332, which is required for interaction with BRCA1, is competent for RPA and Rad51 assembly, indicating that resection is proficient in this background^27^. More recently, the CtIP–BRCA1 complex was found not to be essential for DNA end resection but rather modulated its speed^28^. The resection process is controlled by two core resection machineries in human cells: BLM–DNA2–RPA–MRN and EXO1–BLM–RPA–MRN^29^. DNA resection is also negatively regulated by the HELB helicase in an RPA-dependent manner^30^ and by 53BP1 and RIF1 proteins^31,32^.

Many years ago, we demonstrated that MRE11 and NBS1, which are core components of the early DSB sensing complex MRN, are recruited in a PARP-1-dependent manner to laser-induced DNA damage tracks. MRE11 was further shown to interact non-covalently with PAR via its intrinsically disordered glycine- and arginine-rich region, an interaction that modulates the resection functions of MRE11^10^. We have previously shown that PARP-1 can interact with Ku70 and Ku80^33^ and PARP-1 activity is necessary for Ku binding in Dictyostelium through PAR binding by Ku70^22^. Importantly, PARP-1 activation precedes the recruitment of both the MRN and the Ku complex, previously recognized as the primary DSB sensors that recruit signaling proteins at DSB sites. Because the Ku complex and MRN bind PAR, PARylation may serve to guide and concentrate the Ku and MRN complexes at DSBs to facilitate their loading. As MRN is the initiator of DNA resection while the Ku complex inhibits end resection, we set out to determine whether PARP-1 could affect DNA resection.

The hypersensitivity of HR-deficient cancers to PARP inhibitors (PARPi) provided a conceptual basis for synthetic lethality. PARPi are currently being tested in over 200 clinical studies, with at least 50 trials in phase III (www.clinicaltrials.gov). Because of their specific mechanism of action, PARPi show a low toxicity profile^34^. PARPi has proven to be of significant clinical benefit, even for patients without HR deficiencies. Defining how PARP-1-dependent DNA processing functions mechanistically will help identify genetic markers of sensitivity and resistance to guide PARPi therapy by identifying patients most likely to respond to either single agent or combination therapy through cytotoxic or radiation chemopotentiation^35^. Here, we identify PARP-1 as a critical regulator of DNA end resection of DSBs. We show that PARP-1 recruitment protects DNA ends from nucleolytic degradation and inhibition of PARP-1 leads to hyper-resected DNA double-strand breaks. Our data provide an alternative mechanism by which PARPi function in the presence of irradiation (IR).

## Results

### Recruitment of PARP-1 and PARP-2 at laser-induced DNA breaks

We initially scrutinized the recruitment kinetics of PARP-1 to laser-induced DNA damage. Consistent with previous findings^36,37^, we observed that PARP-1 is recruited rapidly to laser-induced DNA damage tracks within milliseconds (Supplementary Fig. 1). The dynamics of PARP-1 recruitment under normal conditions was compared with the dynamics observed under PARP inhibition with BMN 673 (Talazoparib). The initial rapid accumulation of PARP-1 at sites of damage was followed by a steady reduction over the next 10 min, while in the presence of BMN 673, PARP-1 is lost from the damage site more slowly, possibly due to trapping at DSBs^38^. Similar to PARP-1, PARP-2 retention at laser-induced DNA lesions is normally transient, with a slow decline after an initial maxima at approximately 2 min post damage. However, in cells exposed to BMN 673, an initial rapid accumulation is observed, but rather than decline, there persists a slowly increasing accumulation of PARP-2 over the 10 min experiment (Supplementary Fig. 2). Because the ultrafast recruitment of PARP-1 at DNA lesions precedes and primes the accumulation of other DNA damage repair factors, including the MRN complex, and because PARylation is a determining factor for their local accumulation, we reasoned that PARylation-dependent events might also affect DSB repair pathway choice through DNA end resection.

### Effect of PARPi on DNA end resection

**Fig. 1:**
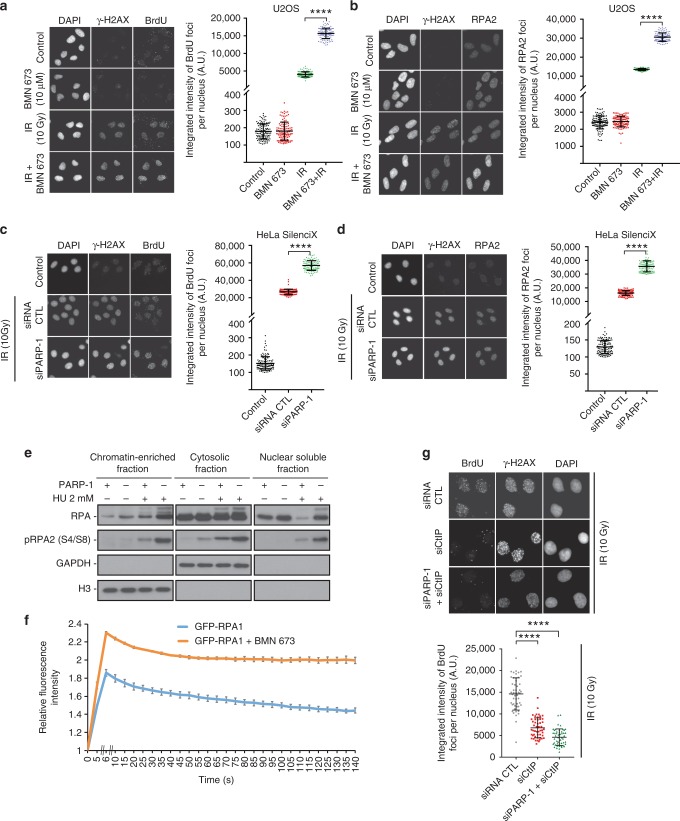
Poly(ADP-ribose) polymerase-1 (PARP-1) regulates DNA end resection and chromatin accumulation of replication protein A (RPA). U2OS cells mock treated, treated with BMN 673, irradiated (10 Gy), or irradiated (10 Gy) in combination with BMN 673 were subjected to immunofluorescence against γ-H2AX, bromodeoxyuridine (BrdU) (**a**), or RPA2 foci formation (**b**). HeLa SilenciX cells underexpressing PARP-1 by siRNA-mediated gene knockdown were subjected to immunofluorescence against γ-H2AX, BrdU (**c**) or RPA2 foci formation after irradiation (10 Gy, 3 h release) (**d**). In panels **a**–**d**, the data show the mean ± s.d. (Mann–Whitney *U* test). **e** HEK293T wild type or CRISPR-PARP-1 were treated with 2 mM hydroxyurea for 16 h, fractionated into chromatin enriched, nuclear soluble and cytoplasmic fractions. **f** GFP-RPA1 recruitment in the absence (blue line) or presence of BMN 673 (orange line). The peak of intensity was at 6 s. Data show the mean ± s.e.m. **g** knockdown of CtIP suppresses the accumulation of single-stranded DNA in HeLa SilenciX PARP-1 knockdown cells after irradiation (10 Gy, 3 h release). The data show the mean ± s.d. ****p ≤ 0.0001 (Mann–Whitney *U* test). Source data are provided as a source data file

We used two different methods to determine whether PARP-1 itself or its catalytic activation influences DNA resection. First, we used a bromodeoxyuridine (BrdU)-based assay for visualizing the single-stranded DNA (ssDNA) product of resection. Second, we measured the accumulation of replication protein A (RPA). RPA is an essential trimeric protein complex that binds to ssDNA in eukaryotic cells. It is recruited to sites of DNA damage when regions of ssDNA are exposed. Hence, it serves as a readout for resection and for ongoing HR. Thus the amount of RPA that accumulates at each site should reflect the amount of ssDNA. Remarkably, both PARP-1 inhibition by BMN 673 or small interfering RNA (siRNA)-mediated PARP-1 silencing led to a substantial increase in the generation of ssDNA as measured by BrdU intensity in U2OS or HeLa cells (Fig. 1a, c). This was further confirmed using CRISPR/Cas9-mediated knockout of PARP-1 in 293T cells (Supplementary Fig. 3A, B). We quantified this data by measuring the total amount of BrdU (or RPA) in each focus. The summed intensity values for each individual focus reveals a greater than two-fold increase in the average amount of ssDNA generated per DSB (focus) when PARP-1 is inhibited by BMN 673 or silenced by siRNA. Consistent with an accumulation of RPA foci following pharmacological inhibition of PARP-1 or PARP-1 knockdown (Fig. 1b, d, Supplementary Fig. 3C), we observed an overall increase of chromatin-bound and phosphorylated RPA (Fig. 1e). Microirradiation experiments also showed enhanced recruitment of GFP-RPA1, GFP-RPA2, or GFP-RPA3 in S-phase cells following pharmacological inhibition of PARP-1 (Fig. 1f, Supplementary Fig. 3E, F). It is well known that the activation of the ATR kinase following perturbations in S-phase relies on a complex mechanism involving ATR recruitment to RPA-coated ssDNA. Consistent with an increase in RPA recruitment in PARP-1 knockdown cells, the activation of ATR was enhanced as judged by using anti-ATR Thr-1989 as a proximal marker of ATR activation^39^ (Supplementary Fig. 3G).

To rule out the possibility that PARP-1 plays an indirect role that promotes the accumulation of ssDNA, we repeated the BrdU-based assay with a knockdown of CtIP, which is expected to suppress DNA resection at DSBs^40^. The simultaneous knockdown of PARP-1 and CtIP completely suppressed the accumulation of ssDNA, implying that knockdown of PARP-1 affects the DSB resection process and does not promote a resection-independent accumulation of ssDNA (Fig. 1g, Supplementary Fig. 4A, B). CtIP foci formation was also increased in PARP-1 knockdown cells (Supplementary Fig. 4C, D). The accumulation of ssDNA was not observed in DNA Ligase IV knockdown cells, suggesting that cells compromised in later stages of NHEJ do not share this phenotype (Supplementary Fig. 4E). Since we are monitoring DNA resection products 3 h after IR, we ascertained that the above results were not a consequence of an accumulation of cells in S/G2 (Supplementary Fig. 5A, B). In addition to the intensity, the number of BrdU or RPA foci per nucleus were increased in BMN 673-treated U2OS cells or PARP-1-silenced HeLa cells compared to controls (Supplementary Fig. 5C–F). Treatment with another PARPi, Veliparib, caused also an enhancement of BrdU or RPA intensity per nucleus (Supplementary Fig. 6A, B).

**Fig. 2:**
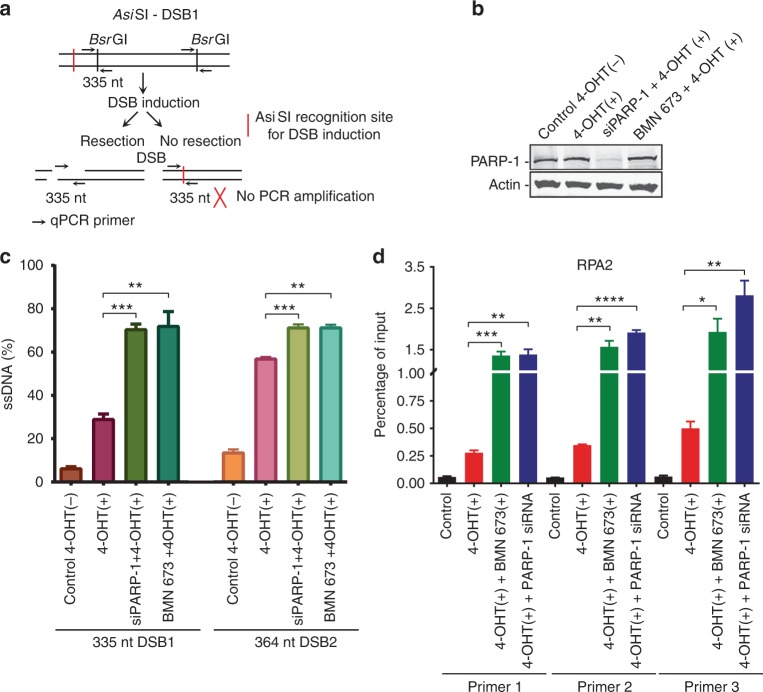
Measurement of double-strand break (DSB) resection in ER-*Asi*SI U2OS cells knocked down for poly(ADP-ribose) polymerase-1 (PARP-1) or treated with a PARP inhibitor. **a** Quantitative polymerase chain reaction (qPCR) primers and probes for measurement of DSB resection at two *Asi*SI sites. **b** ER-*Asi*SI U2OS cells were knocked down for PARP-1 or treated with BMN 673 for 1 h, followed by induction with 300 nM 4-hydroxytamoxifen for 3 h. **c** Quantitation of the percentage of DNA resection. Error bars represent mean ± s.e.m. **p ≤ 0.01, ***p ≤ 0.001 (Mann–Whitney *U* test). **d** Chromatin immunoprecipitation–qPCR was performed with antibody against RPA2 in the ER-*Asi*SI U2OS cells. DSBs were induced at 48–72 h after siRNA transfection or after 1 h BMN 673 treatment. Immunoprecipitated chromatin samples were analyzed by qPCR using specific primer pairs located at Chr 1: chr1_89231183, Chr 6: chr6_90404906, and Chr 21: distal region of chr21_21292316. Error bars represent mean ± s.e.m. *p ≤ 0.05, **p ≤ 0.01, ***p ≤ 0.001, ****p ≤ 0.0001, (Mann–Whitney *U* test). Source data are provided as a source data file

To quantify ssDNA at the sites of DSBs, we used the ER-*Asi*SI system in which the restriction enzyme *Asi*SI is fused to the estrogen receptor hormone-binding domain. Upon treatment with 4-hydroxytamoxifen (4-OHT), the *Asi*SI nuclease translocates to the nucleus and generates up to 150 DSBs at sequence-specific sites^41,42^. In this system, the presence of DSB resection will lead to the generation of ssDNA that cannot be cleaved by the duplex DNA-specific endonuclease *Bsr*GI before PCR. In the absence of DNA resection, the remaining double-stranded DNA (dsDNA) will be cleaved, therefore yielding no PCR products (Fig. 2a). Thus the system can be used to distinguish between ssDNA and dsDNA. Interestingly, PARP-1 depletion or inhibition by BMN 673 (Fig. 2b) led to a ~30% increase in DNA resection compared to the control at two different sites (Fig. 2c). Similarly, PARP-1 inhibition led to a ~3–6-fold increase in bound RPA2 to processed DSBs (Fig. 2d). Altogether, these results show that PARP-1 limits DNA processing in cellulo.

### PARP-1 knockdown cells show decreased 53BP1 and RIF1 foci

**Fig. 3:**
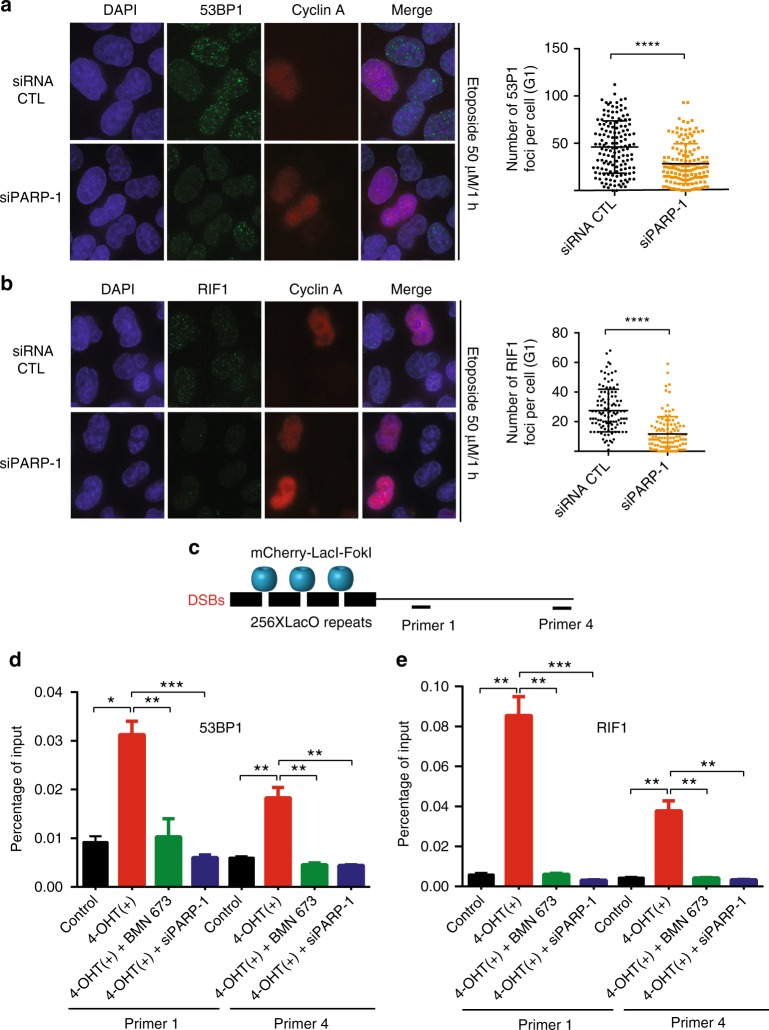
Poly(ADP-ribose) polymerase-1 (PARP-1) inhibition reduces the accumulation of 53BP1 and RIF1 foci. **a** Etoposide-treated HeLa cells were either transfected with an small interfering RNA (siRNA) control or PARP-1 siRNA and subjected to immunofluorescence staining against 53BP1 or Cyclin A. The mean foci count was 46.05 for siRNA CTL and 28.42 for siPARP-1. Data show the mean ± s.d. ****p ≤ 0.0001 (Mann–Whitney *U* test). **b** Etoposide-treated HeLa cells were either transfected with a control siRNA or PARP-1 siRNA and subjected to immunofluorescence staining against RIF1 or Cyclin A. The mean foci count was 27.52 for siRNA CTL and 11.68 for siPARP-1. Data show the mean ± s.d. ****p ≤ 0.0001 (Mann–Whitney *U* test). **c** Cartoon of the U2OS-DSB-reporter with inducible DSB generation by mCherry-*LacI*-*Fok*1. Chromatin immunoprecipitation (ChIP)–quantitative polymerase chain reaction (qPCR) primer sets are shown as p1–p4. ChIP of 53BP1 (**d**) or RIF1 (**e**) from the fixed chromatin of U2OS–DSB reporter cells stably expressing mCherry-*LacI*-*Fok*1 construct and treated with both 0.5 mM Shield-1 (1:500 dilution) and 1 mM 4-hydroxytamoxifen (4-OHT) for 1 h in order to generate DSBs. The ChIP–qPCR primer sets are labeled as primer 1 and primer 4. Data in **d**–**e** show the mean ± s.e.m. *p ≤ 0.05, **p ≤ 0.01, ***p ≤ 0.001 (Mann–Whitney *U* test). Source data are provided as a source data file

The mechanism underlying PARP-1-regulated DNA resection was investigated further. We monitored the accumulation of the resection inhibitors 53BP1 and RIF1 in G1 cells (Fig. 3a, b) depleted of PARP-1. Interestingly, PARP-1 inhibition led to a decrease of 53BP1 and RIF1 foci following etoposide treatment (average number of foci = 46 in the control and 28 in PARP-1 knockdown for 53BP1, and average number of foci = 27 in the control and 11 in PARP-1 knockdown for RIF1). These results were corroborated by chromatin immunoprecipitation (ChIP) analysis using cells stably expressing mCherry-*Lac*I-*Fok*I at an integrated reporter transgene (U2OS-DSB-reporter system (Fig. 3c)), which showed a drastic reduction of 53BP1 (Fig. 3d) or RIF1 accumulation (Fig. 3e) on two different DSBs in PARP-1 knockdown or BMN 673-treated cells.

### PARP-1 blocks DNA resection by MRN-RPA-BLM-EXO1-DNA2

**Fig. 4:**
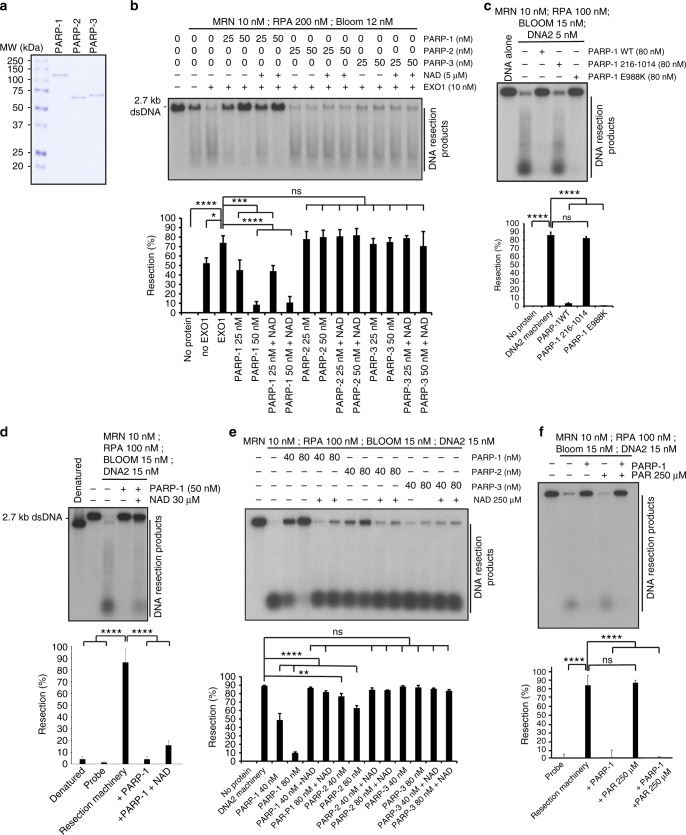
Poly(ADP-ribose) polymerase-1 (PARP-1) limits DNA end resection in vitro. **a** Sodium dodecyl sulfate-polyacrylamide gel electrophoresis of purified human PARP-1, PARP-2, and PARP-3. **b** The indicated PARP proteins were incubated with the MRN-RPA-BLM-EXO1 machinery in the absence or presence of NAD (5 µM). The resection products were detected by autoradiography after agarose gel electrophoresis. Bottom: quantification of the results. **c** The PARP-1 DNA-binding mutant PARP-1 216-1014 fails to inhibit DNA resection. Bottom: quantification of the results. **d** DNA end resection by the MRN-RPA-BLM-DNA2 machinery is decreased in the absence or presence of NAD (30 µM). **e** The indicated PARP proteins were incubated with the MRN-BLM-RPA-DNA2 machinery in the absence or presence of NAD (250 µM). **f** The addition of purified PAR (250 µM) alone does not block DNA resection by MRN-BLM-RPA-DNA2. PARP-1 was used at 87 nM. Error bars in **b**–**f** indicate s.d. from three independent experiments. *p ≤ 0.05, **p ≤ 0.01, ***p ≤ 0.001, ****p ≤ 0.0001 (ordinary one-way analysis of variance). Source data are provided as a source data file

The above data suggest that PARP-1 may be able to directly suppress the activity of DNA resection enzymes. We further examined whether purified PARP proteins (Fig. 4a) could block DNA resection in vitro. We monitored DNA resection of a 3′-end-labeled dsDNA (2.7 kb) via the two main DNA resection machineries MRN-RPA-BLM-EXO1 or MRN-RPA-BLM-DNA2. In the absence of PARPs, the MRN-RPA-BLM-EXO1 assembly resected ~75% of the 2.7 kb substrate (Fig. 4b). When the reaction was supplemented with PARP-1, a concentration-dependent inhibition was observed. At 50 nM PARP-1, only 10% of the DNA could be resected within the 60 min incubation time, without NAD or at a NAD concentration that still supports DNA binding. As specificity controls, we also performed similar reactions with PARP-2 and PARP-3. Importantly, PARP-2 and PARP-3 enzymes did not inhibit MRN-RPA-BLM-EXO1-mediated DNA degradation. PARP-1 also blocked the MRN-RPA-BLM-DNA2 complex and PARP-1-mediated inhibition was retained with the catalytic mutant E988K but abolished when a PARP-1 fragment devoid of its zinc fingers (PARP-1 216-1014) was used (Fig. 4c, Supplementary Fig. 6C). PARP-1 216-1014 has been reported to have a severely decreased affinity (250-fold less) for DNA lesions compared to wild-type (WT) PARP-1^23^. PARP-1 blocked the resection complexes at 5 and 30 µM concentration of NAD^+^, where PARP-1 undergoes only moderate PAR automodification (Supplementary Fig. 6D, E) and remains bound to DNA (Fig. 4b–d, Supplementary Fig. 6F, G). In contrast, the higher NAD^+^ concentration of 250 µM NAD^+^, where PAR automodification releases PARP-1 from DNA, prevented PARP-1 inhibition of resection (Fig. 4e, Supplementary Fig. 6F, G). Since even low levels of PAR alone could sequester protein components of the resection reaction leading to suppression of DNA resection, we performed reactions with protein-free PAR (Fig. 4f). This failed to inhibit DNA resection. These results show that PARP-1 but not PARP-2, PARP-3, or PAR can robustly inhibit DNA resection through a direct DNA-binding mechanism.

**Fig. 5:**
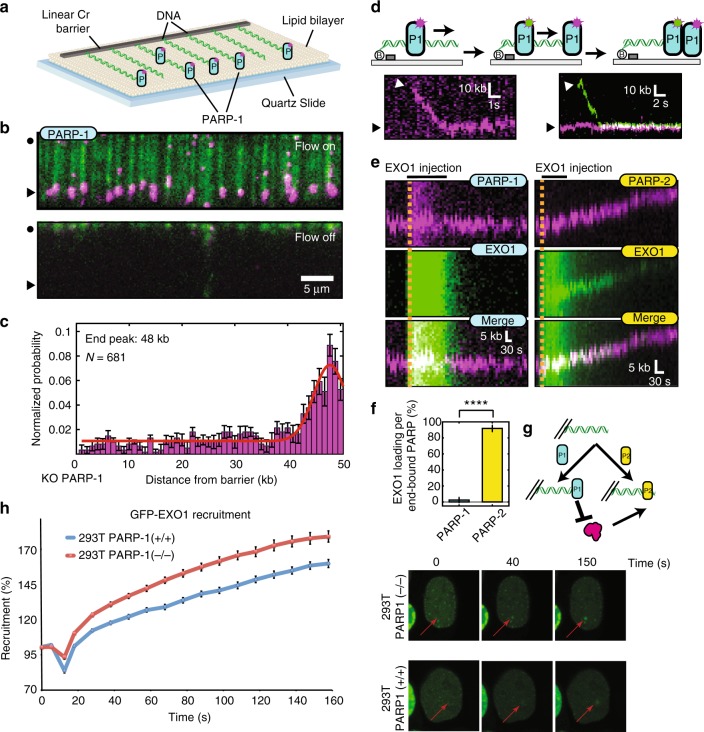
Poly(ADP-ribose) polymerase-1 (PARP-1) slides to accumulate at ends and prevent EXO1 binding. **a** Illustration of the DNA curtain assay with fluorescent PARP-1 (blue ovals). **b** Fluorescent PARP-1 (magenta) binding to DNA curtains in the presence (top) or absence (bottom) of buffer flow. Black circles indicate the diffusion barrier and arrows indicate the DNA ends. After PARP-1 was loaded, the DNA was stained with the fluorescent intercalating dye YoYo-1. **c** Histogram of the positions of 681 individual PARP-1 molecules along the length of the DNA substrate. Error bars were calculated by bootstrapping the data^74^ and indicate a 70% confidence interval. **d** Illustration (top) and kymograph (bottom) of two differently labeled PARP-1 (P1) molecules binding to the DNA end. PARP-1 reached the end by buffer flow-assisted one-dimensional sliding 88% of the time (*N* = 109/124). **e** Representative kymographs of EXO1 resection of DNA pre-bound with PARP-1 (left) or PARP-2 (right). **f** Quantification of EXO1 loading events per DNA end bound by PARP-1 (left; 2.4 ± 3.4%; *N* = 42 PARP-1 molecules) or PARP-2 (right; 91.8 ± 4.1%; *N* = 65 PARP-2 molecules). Error bars represent mean ± s.e.m. ****p ≤ 0.0001, (Student’s *t* test). **g** Model for PARP-1 inhibition of EXO1. P2 refers to PARP-2. YoYo-1 was omitted from all experiments described in **c**–**f**. **h** Recruitment of GFP-EXO1 at laser-induced double-strand breaks in HEK293T wild-type or PARP-1−/− cells. Source data are provided as a source data file

We used a high-throughput single-molecule DNA curtain assay to directly observe how PARP-1 inhibits EXO1 processing of substrate DNA. Arrays of DNA molecules (48.5 kb, derived from λ-phage) were assembled on the surface of a microfluidic flowcell coated with a surface-passivating fluid lipid bilayer^43^. The DNA substrate was linked to the bilayer via a biotin–streptavidin linkage. Fluorescently labeled PARP-1 was injected into the flowcell and visualized using total internal reflection fluorescence microscopy on thousands of DNA molecules for high-throughput data collection and analysis (Fig. 5a). Nearly all PARP-1 molecules were bound to the end of the DNA. This conclusion is based on the observation that turning off buffer flow led to the coordinated retraction of both the DNA and associated PARP-1 molecules to the diffusion barrier (Fig. 5b, c). Most PARP-1 molecules localized to the DNA end via binding internally and sliding along the DNA in the direction of buffer flow (88% of PARP-1 molecules, *N* = 109/124). This indicates that PARP-1 is able to diffuse along the DNA and one-dimensional (1D) diffusion may represent a mechanism by which PARP-1 quickly associates with DNA ends or other DNA lesions. In conditions where PARP-1 was pre-bound to the end of DNA molecules (Fig. 5d, magenta in right panel), a second PARP-1 molecule (Fig. 5d, green in right panel) bound upstream and slid along the DNA to co-localize at the DNA end. Consequently, the red and green traces can be seen to merge in the kymograph (Fig. 5d). Pre-bound PARP-1 blocked DNA resection by preventing EXO1 loading onto the DNA end (Fig. 5e–g). This suggests that PARP-1 may physically occlude the end of DNA. In contrast, end-bound PARP-2 did not block EXO1 loading and DNA resection. Consistent with this, microirradiation experiments showed enhanced accumulation of GFP-EXO1 in PARP-1-deficient cells at laser-induced DNA damage sites (Fig. 5h) and phosphorylated EXO1 accumulated more in the absence of PARP-1 (Supplementary Fig. 7A). Altogether, our results show that PARP-1 counteracts DNA resection in vitro, likely by occluding the free DNA ends from the EXO1 or DNA2 resection machineries.

### PARP-1 is required for efficient loading of the Ku complex

**Fig. 6:**
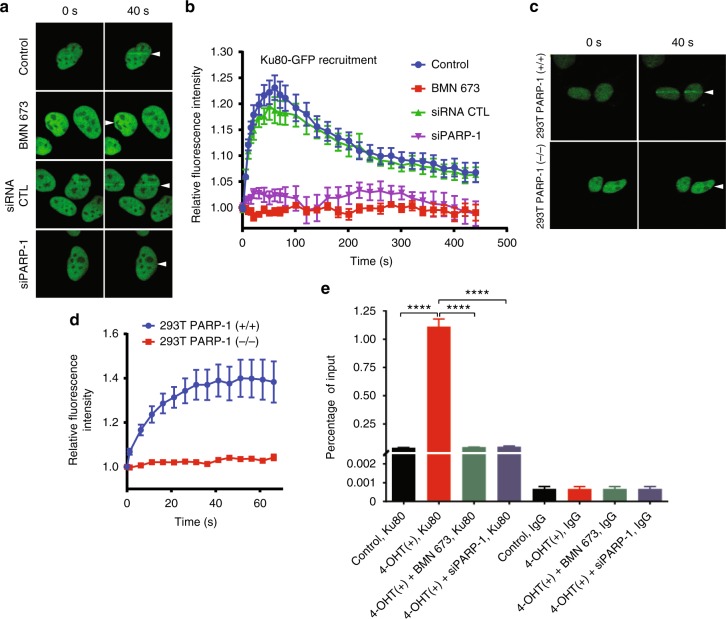
Poly(ADP-ribose) polymerase-1 (PARP-1) knockdown or pharmacological inhibitor affects Ku80 recruitment on double-strand breaks. **a** U2OS cells stably expressing the Ku80-GFP were mock treated, treated with BMN 673, or silenced with PARP-1 siRNA and subjected to laser microirradiation-track studies. **b** Quantification of the relative fluorescence intensity of Ku80-GFP over time. Error bars represent mean with s.e.m. **c** Recruitment of Ku80-GFP in PARP-1-deficient CRISPR 293T cells is severely affected. **d** Quantification of the relative fluorescence intensity of Ku80-GFP over time. Error bars represent mean with s.e.m. **e** AsiS1-ER-U2OS reporter cells were cultured and treated with 4-hydroxytamoxifen (300 nM) for 1 h. Soluble chromatin fractions were prepared and subjected to immunoprecipitation against IgG control and Ku80-specific antibody. Immunoprecipitated chromatins fractions were analyzed by quantitative polymerase chain reaction using specific primer pairs: chr1_89231183. Error bars represent mean ± s.e.m. ****p ≤ 0.0001 (Mann–Whitney *U* test). Source data are provided as a source data file

Another mechanism whereby PARP-1 could inhibit end resection is through regulation of the association of the Ku complex with DNA ends. Consequently, we performed laser microirradiation experiments to determine whether Ku80-GFP recruitment to sites of DNA damage is sensitive to PARPis (Fig. 6a, b). We found that the recruitment of Ku80-GFP was almost completely inhibited in cells treated with PARPi but robustly recruited in control cells. We next tested PARP-1-deficient cells and found that they also fail to efficiently recruit Ku80-GFP to sites of DNA damage (Fig. 6c, d). In order to determine the influence of PARP activity on the association of Ku80 with DNA ends, we performed ChIP experiments in the presence or absence of PARP-1 or PARPi (Fig. 6e) and in the presence of RNAse to avoid indirect binding through RNA. We find that Ku association with the DSB is dependent on both PARP-1 and PARP-1 activity. Consequently, in addition to acting as a direct inhibitor of DNA end resection, PARP-1 also inhibits end resection by promoting Ku loading onto the DSB. To assess this further on other NHEJ components, we monitored phospho-DNA-PKcs foci formation in PARP-1 U2OS CRISPR/Cas9-mediated knockout cells. Consistent with our previous studies showing a decrease of NHEJ in cellulo following ABT-888 treatment^44^, a decrease of phosphorylated DNA-PKcs foci was observed (Supplementary Fig. 7B). A Ku80 knockdown in HEK 293T cells leads to enhanced EXO1 recruitment (Supplementary Fig. 7C), suggesting that the recruitment of Ku80 by PARP-1 is a critical event for regulating EXO1-mediated DNA resection.

### Increased resection tracks and HR in PARP-1-deficient cells

**Fig. 7:**
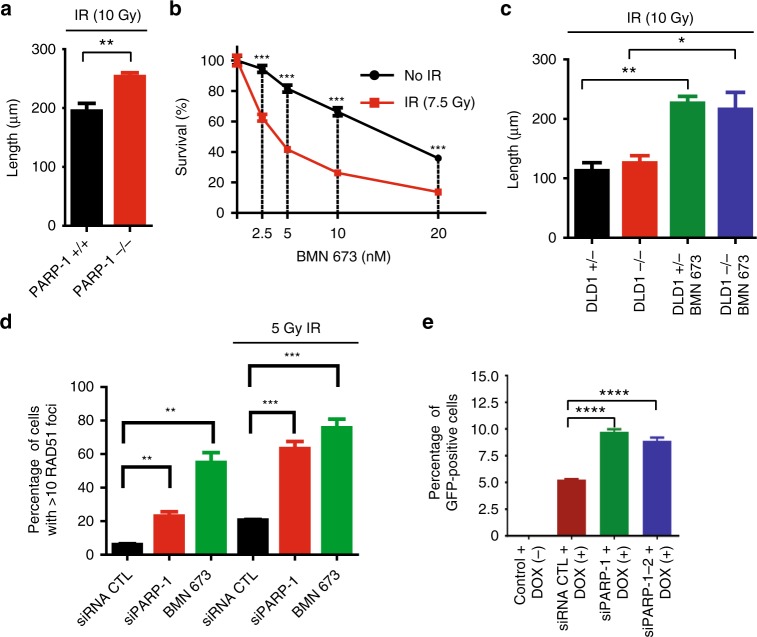
Single-molecule analysis of resection tracks (SMART) analysis and effect of poly(ADP-ribose) polymerase-1 (PARP-1) on homologous recombination. **a** SMART of PARP-1-deficient mouse embryonic fibroblasts (MEFs). MEFs were treated with 10 Gy irradiation (IR). Error bars represent mean ± s.e.m. **b** Treatment of HeLa cells with BMN 673 results in IR sensitivity. Error bars represent mean with s.e.m. **c** SMART of BRCA2-proficient (DLD1 BRCA2 (+/−)) and BRCA2-deficient cells (DLD1 BRCA2 (−/−)) either mock treated of treated with BMN 673 (5 μM) were irradiated (10 Gy). Error bars represent Mean with s.e.m. **d** Attenuation of PARP-1 increases RAD51 foci in cellulo. Error bars represent mean ± s.e.m. **e** Homologous recombination is increased in PARP-1 knockdown cells. Error bar represents mean ± s.d. *p ≤ 0.05, **p ≤ 0.01, ***p ≤ 0.001, ****p ≤ 0.0001 (Mann–Whitney *U* test). Source data are provided as a source data file

Next, we measured DNA resection in cellulo using a high-resolution technique to see whether PARP-1 inhibition would lead to increased resection in different genetic contexts. The single-molecule analysis of resection tracks (SMART) assays is a very sensitive technique that can detect DNA resection upon IR, while almost no fibers can be detected without DNA damage^28^. It has been reported that BRCA1 is important to control the speed of DNA resection^28^ and that inhibition of 53BP1 in BRCA1-deficient cells rescue these cells through enhanced DNA resection^45^. Hence, our data predict that the DSB-induced over-resection phenotype would not appear in BRCA1-deficient cells when challenged with the PARP-1 inhibitor BMN 673. However, the DNA resection machinery is intact in BRCA2-deficient cells, because BRCA2 acts later in HR, and we should observe this over-resection phenotype under the same conditions. First, SMART assays revealed that PARP-1-deficient cells have longer resection tracks after IR treatment (Fig. 7a) and are IR sensitive in survival assays (Fig. 7b). When cells were treated with BMN 673 and IR, BRCA1 knockdown led to a decrease of BrdU accumulation, which could be partially rescued by 53BP1 knockdown (Supplementary Fig. 8A, B, D). SMART assays also recapitulated these results (Supplementary Figs. 8C, D and 9A). Conversely, BRCA2 knockdown or DLD1 BRCA2 (−/−) cells showed a similar increase of BrdU staining (Supplementary Fig. 8A, B) or DSB resection tracks following treatment with BMN 673 (Fig. 7c) compared to the controls. These results show that PARP-1 inhibition-mediated over-resection of DSBs is achieved when cells have an effective DNA resection machinery.

Based on these results, we hypothesize that PARP-1 plays an important regulatory role in the DNA damage response by influencing DNA resection and consequently DNA repair. HR depends heavily on the extent of DNA resection. To address whether PARP-1 influences HR, we monitored RAD51 foci formation following γ-irradiation (Fig. 7d). In agreement with previous findings with an older generation of PARP-1 inhibitors^46^, we observed that the percentage of cells harboring >10 RAD51 foci was increased in HEK 293T cells subjected to CRISPR/Cas9-mediated knockout of PARP-1 (Supplementary Fig. 9B, C) or in cells treated with a siRNA targeting PARP-1 or exposed to PARP-1 inhibition (Fig. 7d). In addition, using a doxycycline (Dox)-inducible I-*Sce*I/DRGFP cell line, HR was increased with PARP-1 knockdown (Fig. 7e), BMN 673, or ABT 888-treated cells (Supplementary Fig. 9D). Collectively, these results show that PARP-1 negatively regulates HR in cellulo.

## Discussion

In this study, we show evidence that PARP-1 antagonizes the activity of the MRN-RPA-BLM-EXO1 and MRN-RPA-BLM-DNA2 machineries for DNA DSB repair. Interestingly, PARP-1 mediates this effect through DNA end binding and promoting Ku80 loading. Furthermore, loss of PARP-1 leads to a decrease in the accumulation of HR suppressors 53BP1 and RIF1 at DSBs, which in turn increases the DNA accessibility to EXO1 resulting in excessive degradation of DNA lesions. Such an effect can be obtained with either PARP-1 knockdown or pharmacological inhibition of PARP-1 activity. Thus mammalian cells have evolved several distinct regulatory systems that limit ssDNA overhang formation. First, a PARP-1-dependent mechanism influencing the Ku heterodimer and the 53BP1 pathway, and second, HELB that limits end resection in an RPA-dependent manner^30^. Recent studies have shown that DYNLL1^47^ and the Shieldin complex^48–50^ can also counteract DNA resection.

**Fig. 8:**
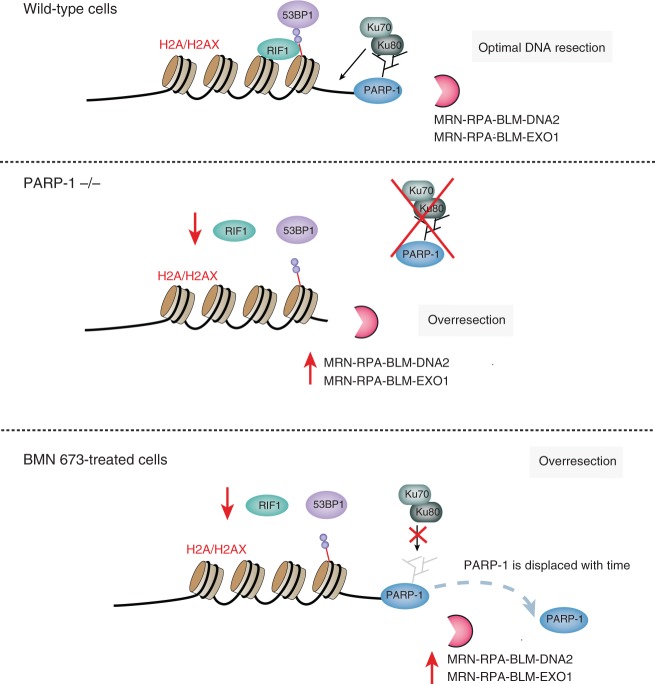
Model. Following DNA damage, poly(ADP-ribose) polymerase-1 inhibition leads to a decrease of Ku80-end protection, RIF1 and 53BP1 foci formation, and increased DNA resection. Details are given in the text

PARP-1 is an abundant nuclear chromatin-associated protein, well characterized for its high DNA damage-sensing ability. Once encountering free DNA ends, PARP-1 is catalytically activated and generates large amounts of PAR, which can function as a scaffold for the recruitment of a variety of DNA repair proteins^12^. It has been proposed that the local accumulation of PAR at DNA damage sites promotes liquid demixing, a phase separation event leading to compartmentalization of repair foci^12^. PAR polymers not only provide a loading platform for DDR-associated proteins and repair factors but also reprograms their functions through spatial and temporal interactions with their PAR reading motifs^12,44^. We envision that PARP-1 activation orchestrates the initial steps of DNA resection, granting access to the resection machineries. PARP-1 interacts with DNA-PKcs/Ku70/Ku80^51^ and mediates this effect through DNA end binding and recruitment of the Ku complex to DNA ends. Although Ku and PARP-1 have been found to compete for binding to DNA end in vitro^52^, temporally, PARP-1 precedes Ku loading and its activity is required to load Ku onto DSB ends. This correlates well with the timing of PARP-1-mediated displacement of histones^9^, suggesting that PARP-1 activity is necessary to prepare chromatin for loading of Ku onto broken DSB ends. When PARP-1 is absent, neither PARP-1 nor Ku assemble to protect the DNA ends when analyzed by ChIP at nuclease-induced DSBs. Consequently, EXO1 has higher access to DSBs, and with a concomitant decrease of RIF1 and 53BP1, this leads to excessive DNA processing. In line with this observation, using the ER-*Asi*SI system, the group of Tanya Paull has shown that siKu86 or Ku86 conditional HCT116 cells show increased DNA resection^42^. This enhanced DNA resection has been observed in PARP-1 knockdown and PARP-1 knockout using SMART analysis, RPA staining as a surrogate marker of ssDNA accumulation, and native anti-BrdU staining. It is important to note that this excess of ssDNA is dependent on CtIP thereby confirming that the ssDNA detected is generated by DNA end resection. In a similar manner, we also observed excessive DSB processing in Veliparib- or BMN 673-treated cells. Initially, BMN 673 delays the displacement of PARP-1 on DSBs but does not prevent displacement, which reaches completion over a 10 min time frame following laser-induced DNA damage. At that point, it will no longer protect DSB ends, leading to a similar phenotype as a complete deletion of PARP-1 (Model in Fig. 8).

Using purified proteins, we also show that PARP-1 directly blocks both the EXO1 and DNA2 end resection machineries. Specifically, PARP-1 is able to slide to the end of DNA. This suggests a search mode employing 1D diffusion, which should stimulate the rate of recognition of newly formed DNA ends relative to three-dimensional diffusion mechanisms. Using single-molecule microscopy, we determined that PARP-1, but not PARP-2, prevents the binding of EXO1 to DNA ends. The structural differences that allow PARP-1 but not PARP-2 or PARP-3 to inhibit end resection would be interesting to determine, given that PARP-1 physically occludes DNA ends from recognition by EXO1. The regulatory zinc finger domains that are unique to PARP-1 are likely to be key to this specificity of function. Our data suggest that, even prior to Ku loading, which strongly prevents the loading of EXO1, PARP-1 acts to block the end resection machinery. This observation fits into a model where both PARP-1 and Ku limit end resection, possibly by controlling the accumulation of the MRN complex^53^ and CtIP and that loss of either PARP-1 or Ku binding results in over-resection. We propose that this mechanism is conserved in pluricellular organisms, as the Iliakis group report that Chinese hamster ovary (CHO) Hamster cells treated with BMN 673 also have elevated DNA processing as measured by RPA foci^54^. PARP-2 has recently been shown to promote DNA resection^55^, but since BMN 673 inhibits PARP-2^56^, it might not contribute to the over-resection phenotype observed in BMN673-inhibited cells.

Our work provides a conceptual framework to explain many observations reported in previous studies detailing the effects of PARP inhibition in a variety of contexts involving DNA recombination transactions. Since the early 1980s, PARP-1 has been proposed to carry out an antirecombinogenic function. The group of Oikawa et al. first described a positive correlation between PARPis (benzamide and *m*-aminobenzamide) and induction of sister-chromatid exchanges (SCEs) in CHO-K1 cells^57^. Hori demonstrated that reduced NAD as well as inhibition of PARP-1 (using 3-aminobenzamide) lead to a significant increase in SCEs in CHO cells^58^. Morrison et al. using PARP-1^−/−^ mice, provided evidence of PARP-1 functions in maintaining genomic stability by demonstrating that PARP-1 is an anti-recombinogenic factor that inhibits ligation between the DNA termini exposed during (V(D)J) recombination^59^. More recently, it was shown that PARP-1 PARylates BRCA1, and short- and long-track gene conversions, as well as chromosome aberrations after DNA damage, were increased by a BRCA1 PARylation mutant. In addition, treatment with olaparib also led to an enhancement of both types of HR frequencies^60^. Altogether, our data suggest that loss of PARP-1 facilitates HR, through enhanced DNA resection accounting for the (i) increase of sister chromatid exchanges^57^; (ii) anti-recombinogenic function of PARP-1^59^; and (iii) increased HR repair by a BRCA1 PARylation mutant^60^. PARP-1 inhibition-induced HR is in accordance with our previous findings with another PARPi, ABT-888, which remained unexplained at the time^44^. It also mimics the effect of a HELB knockdown, another DNA resection inhibitor^30^.

One of the first models proposed to explain the antitumor effects of PARPis in HR-deficient cells was based on the functions of PARP-1 in BER. This model postulated that catalytic inhibition of PARP-1 results in the accumulation of single-strand breaks that could not be repaired in HR-deficient cells. Two observations challenged this model. First, it was not possible to demonstrate increased single-strand breaks after PARP inhibition^61^, and synthetic lethality was not achieved when XRCC1, a key BER protein, was downregulated in BRCA2-deficient cells^62^. Hence, these observations raised the possibility that the effects of PARPis may be mediated through a mechanism distinct from BER. Consistent with this, Patel et al. have shown that deregulated NHEJ plays a major role in generating the genomic instability and cytotoxicity in HR-deficient cells treated with PARPis^62^.

We suggest that the observed synthetic lethality and cytotoxicity in different genetic contexts can be related to aberrant DNA resection as consequence of PARP-1 inhibition and DNA damage. Under these conditions, this phenotype will only be attained if DSBs are created and the DNA resection machinery is functional. This conclusion is highlighted by SMART analysis or BrdU staining of BRCA1- and BRCA2-deficient cells. Recent phase III studies have shown that PARPi activity extends beyond BRCA-related cancers for ovarian cancers devoid of known *BRCA* mutations, especially when platinum sensitivity and high-grade serous histology are present^63^. We propose that this effect could be due to misregulation of DNA resection. There are several ongoing clinical trials combining PARP-1 inhibitors with radiation therapy for which our study provides mechanistic insights into the tumor-killing activity observed in the clinic. Collectively, our results highlight that the functionality of DNA resection enzymes in response to DNA damage may be an important criterion to consider for the cell’s ability to survive BMN 673 in the presence of DNA damage during clinical interventions in breast/ovarian cancer and other solid tumors.

## Methods

### Cell lines, cell culture, drugs, and DNA constructs

Mouse embryonic fibroblasts proficient for PARP-1 (WT), or deficient for PARP-1 (PARP-1 (−/−)) were cultured in Dulbecco’s modified Eagle’s medium (DMEM) supplemented with 10% fetal bovine serum (FBS) (Hyclone-ThermoFisher Scientific, Ottawa, Canada). U2OS, HeLa, and HeLa PARP-1 SilenciX control (Tebu-bio) were cultured in DMEM with 10% FBS. PARP-1 HeLa SilenciX is a cell line engineered to stably knock down PARP-1 via RNA interference. Cells were maintained under hygromycin B selection (250 μg/mL; Invitrogen). U2OS-PARP-1 (−/−) cells were cultured in DMEM supplemented with 10% FBS and 2 μg/mL puromycin. U2OS cells stably expressing GFP-RPA2 were maintained in DMEM through continuous G418 selection (500 μg/mL; Invitrogen). The ER-*Asi*SI U2OS cell line was maintained in phenol red-free DMEM media supplemented with 10% charcoal-stripped FBS (Sigma) and 1 μg/mL puromycin. In order to induce DNA damage, AsiS1 U2OS cells were treated with 300 nM 4-OHT for 3 h. U2OS cells stably expressing an mCherry-LacI-FokI construct containing an integrated reporter transgene were maintained in DMEM by puromycin (2 μg/mL) and hygromycin B (100 μg/mL) selection. To induce DNA DSBs, cell lines were treated with both 0.5 mM Shield-1 and 1 mM 4-OHT for 1 h. Human embryonic kidney 293 cells (HEK 293), HEK 293T, or HEK 293T-PARP-1 (−/−) cells were cultured in DMEM supplemented with 10% FBS.

### Generation of PARP-1 CRISPR/Cas9

U2OS cells were transfected with the appropriate guide RNA against PARP-1, cloned in a pSpCas9(BB)-2A-Puro (PX459) V2.0. The sgDNA sequence (5′-CGATGCCTATTACTGCACTG -3′) was cloned at the *Bbs*I site into pSpCas9(BB)-2A-Puro (PX459) V2.0 (Addgene plasmid ID: 62988). The positive clone was confirmed by restriction enzyme digestion (*Bbs*I and *Age*I) of purified plasmids to check for insertion and further confirmed by Sanger sequencing. Twenty-four hours later, the transfected cells were selected in medium containing 2 µg/mL puromycin and then subcloned into 96-well plates. Once at sufficient cell density, the subclones were analyzed for the presence of the target protein by western blotting (PARP-1).

### Cell fractionation

Cell fractionation was carried out as described in ref. ^64^ with slight modifications. Briefly, 3 × 10^6^ HEK293T or CRISPR PARP-1 (−/−) cells per condition were collected and resuspended in 200 µL of buffer A (10 mM HEPES pH 8.0, 10 mM KCl, 1.5 mM MgCl_2_, 0.34 M sucrose, 10 % glycerol, 1 mM dithiothreitol (DTT), 1 mM phenylmethanesulfonylfluoride (PMSF), 0.1 % Triton-X-100, 10 mM NaF, 1 mM Na_3_VO_4_, supplemented with protease inhibitors) and kept for 5 min on ice. The soluble cytoplasmic fraction (S1) was separated from the nuclei (P2) by centrifugation for 4 min at 1300 × *g* at 4 °C. The nuclear fraction P2 was washed twice with 300 µL buffer A, then resuspended in 200 µL buffer B (3 mM EDTA, 0.2 mM EGTA, 1 mM DTT, 1 mM PMSF, 10 mM NaF, 1 mM Na_3_VO_4_, and protease inhibitors) and kept for 30 min on ice. The insoluble chromatin fraction (P3) was separated from nuclear soluble proteins (S3) by centrifugation for 4 min at 1700 × *g* at 4 °C and washed three times with solution B. S1 was cleared of insoluble proteins by centrifugation at 14,000 × *g* for 15 min at 4 °C and the supernatant (S2) was kept for analysis. Cell fractions were subsequently analyzed by western blotting.

### Antibodies, reagents, resources, and siRNAs

The antibodies used in this study as well as the working dilutions are listed in Supplementary Table 1. Key reagents or resources are listed in Supplementary Table 2. siRNAs are listed in Supplementary Table 3.

### Western blot analysis

Total cell lysates were prepared by lysing cells in RIPA buffer (25 mM Tris–HCl, 125 mM NaCl, 1% Nonidet-P-40, 0.5% sodium deoxycholate, 0.1% sodium dodecyl sulfate (SDS), and a complete protease inhibitor cocktail (Roche)). Equal amounts of total protein were separated by SDS–polyacrylamide gel electrophoresis and then transferred to polyvinylidene difluoride membrane (BioRad) and immunoblotted with antibodies (Supplementary Table 1). When secondary antibodies conjugated with infrared-specific dyes (either Alexa Fluor 680 or Alexa Fluor 750) were used, fluorescence was imaged on the Odyssey Infrared Imaging system (LiCor Biosciences).

### Transfection and siRNA

Transient siRNA transfections were carried out with Lipofectamine RNAiMax (Invitrogen) or Oligofectamine (Thermo Fisher Scientific) and analyses were performed 48–72 h after siRNA transfection. The siRNAs used in this study can be found in Supplementary Table 3.

### Immunofluorescence staining

The effect of PARP-1 knockdown on IR-induced DNA end-resection was analyzed by immunofluorescence staining against RPA2 and BrdU. For RPA2 immunodetection, cells were pre-extracted with RPA buffer (25 mM HEPES pH 7.9, 300 mM sucrose, 50 mM NaCl, 1 mM EDTA, 3 mM MgCl_2_, and 0.5% Triton X-100) for 5 min on ice before being fixed at the indicated incubation time points after IR. This method removes nucleoplasmic signal and helps in the detection of foci. Cells were washed two times with phosphate-buffered saline (PBS) followed by fixation with 4% paraformaldehyde (w/v) in PBS for 15 min at room temperature. After two washes with PBS, cells were permeabilized in 0.5% Triton X-100 in PBS for 5 min. Cells were co-stained with primary antibodies against γ-H2AX (Active motif) and RPA2 (Abcam) in PBS for 1 h at room temperature. After three washes with PBS, cells were stained with goat anti-rabbit Alexa Fluor 488 and anti-mouse Cy3 secondary antibody (Molecular Probes, 1:400) in PBS for 30 min at room temperature.

CtIP and Geminin immunofluorescence was performed as reported previously^65^. For RIF1, 53BP1, and cyclin A immunofluorescence staining, cells were either untreated or treated with 50 μM etoposide for 1 h and fixed with 4% paraformaldehyde in PBS for 15 min. Then cells were washed with TBS and fixed with cold methanol (−20 °C) for 5 min and permeabilized with PBS containing 0.2% Triton X-100 for 5 min and washed three times 5 min with TBS. The cells were quenched with 0.1% sodium borohydride for 5 min, washed once with TBS, blocked in PBS containing 10% goat serum and 1% bovine serum albumin (BSA) for 1 h, and incubated with the primary antibody diluted in PBS 1% BSA for 2 h at room temperature. Coverslips were washed three times for 10 min with TBS before 1-h incubation with the appropriate secondary antibody conjugated to a fluorophore. Cells were rinsed again three times for 10 min with TBS. Coverslips were mounted onto slides with ProLong Gold (Thermo Fisher Scientific) antifade mountant with 4’,6-diamidino-2-phenylindole (DAPI) (Life technologies).

For RAD51 and phosphoDNA-PKcs(S2056) immunostaining, cells were either untreated or treated with 5 Gy IR, released for 1 h, and fixed with 4% paraformaldehyde in PBS for 25 min. Next, cells were permeabilized with PBS containing 0.5% Triton X-100 (PBS-T) for 15 min and washed three times with PBS 1×. The cells were blocked in PBS containing 10% FBS for 1 h and incubated with the primary antibody (RAD51: 1:1000 or phosphoDNA-PKcs(S2056): 1:500) diluted in the blocking buffer for 2 h at room temperature. Coverslips were washed three times with PBS before 1-h incubation with the appropriate secondary antibody (1:1000) conjugated to a fluorophore again in blocking buffer. Cells were rinsed twice with PBS 1×, then incubated in (1:1000) PBS-DAPI solution for 5 min, and then washed twice with PBS 1×. Coverslips were mounted onto slides with ProLong Gold antifade mountant.

### Recruitment of RPA and EXO1 to laser-induced DNA damage sites

The evaluation of the recruitment kinetics of RPA to DNA damage sites was performed essentially as described^6^ with the exception of the following modifications. After overnight transfections with Effectene reagent (Qiagen), unsynchronized HEK 293 cells expressing the indicated RPA subunit fused to green fluorescent protein (GFP) and mCherry-PCNA fusion protein were incubated with fresh medium containing 1 μg/mL of Hoechst 33342 for 15 min at 37 °C and treated with 5 µM of PARPi BMN 673 (20 mM stock solution prepared in dimethyl sulfoxide (DMSO), Selleckchem) 1 h prior to microirradiation and recruitment analysis. A 37 °C pre-heated stage with 5% CO_2_ perfusion was used for time-lapse analysis on a Zeiss LSM-510 META NLO laser-scanning confocal microscope (×40 objective). Localized DNA damage was generated along a defined region across the nucleus of a single living cell by using a bi-photonic excitation of the Hoechst 33342 dye, generated with a near-infrared 750-nm titanium:sapphire laser line (Chameleon Ultra II, Coherent Inc.) The laser output was set to 1.5% with 5 iterations. For each cell, 30 images were collected with a 5 sec interval. A Multi-Time macro developed in-house for the AIM software v3.2 (Zeiss) was used for image acquisition. Background and photobleaching corrections were applied to each dataset as described^10^. The average accumulation ± s.e.m. of RPA was plotted using a minimum of ten recruitment kinetic profiles per each RPA construct from three independent experiments. Only S-phase-positive PCNA (proliferating cell nuclear antigen) cells were chosen for recruitment^66^. Recruitment of GFP-EXO1 to laser-induced DSBs was performed as reported previously^67^.

### Recruitment of Ku80 to laser-induced DNA damage sites

For Ku80 laser microirradiation experiments, a 1-µm diameter band of damage was introduced across the width of the nucleus. Background was determined based on measuring the fluorescence intensity outside of the cells (i.e., in regions containing only the growth medium). Fluorescence loss due to photobleaching that takes place during acquisition is removed by normalizing the total nuclear fluorescence to remain constant throughout the experiment. The fluorescence intensity of the damaged region was then monitored over time after correcting for background and fluorescence loss. The distribution of recruited protein can deviate from the initial band for two reasons. First, the distribution of chromatin determines whether or not the full width and full diameter of the band are sites of DNA damage. Second, as we have previously published, there is a decondensation of the damaged chromatin that causes the band to expand in width beyond the boundary of the original damaged area. Consequently, measurements restricted to the 1-µm wide band where the laser microirradiation took place will slightly underestimate the extent of recruitment and chromatin-bound proteins that are unaffected will commonly show a very slight decrease in fluorescence intensity within the region.

### BrdU/ssDNA assays

Cells were pre-incubated in the presence of 10 μM BrdU (Sigma) for 16 h followed by a 3-h incubation after IR at 10 Gy. Cells were subjected to in situ fractionation on ice for 10 min using sequential extraction with two different buffers. Pre-extraction buffer 1 (10 mM PIPES, pH 7.0, 300 mM sucrose, 100 mM NaCl, 3 mM MgCl_2_, and 0.5% Triton-X100) and followed by pre-extraction buffer 2 (10 mM Tris pH 7.5, 10 mM NaCl, 3 mM MgCl_2,_ 1% Nonidet P-40, and 0.5% sodium deoxycholate). Cells were washed three time with PBS followed by fixation with 4% paraformaldehyde (w/v) for 15 min at room temperature. Cells were washed with PBS and permeabilized in 0.5% Triton X-100 in PBS for 5 min. Cells were incubated overnight at 4 °C with anti-BrdU antibody under non-denaturing conditions. In these native conditions, the anti-BrdU antibody only has access to its epitope in ssDNA. Unbound primary antibody was removed by washing in PBS at room temperature followed by incubation with the anti-mouse Cy3 secondary antibody for 30 min at room temperature. Slides were then washed for four times in PBS before mounting with Vectashield mounting medium (Vector Laboratories) containing DAPI. BrdU foci were observed by using an upright fluorescence microscope (Zeiss AxioImager.Z1) with a Plan Neofluar 1.3 N.A. ×40 oil immersion objective. Image analysis was carried out by the ImageJ software (version 1.51k). The integrated intensity of individual BrdU foci and RPA2 foci were quantified by using GDSC ImageJ Find Foci plugins^68^.

### ChIP assays

The effect of PARP-1 knockdown and PARP inhibition on RPA2, 53BP1, and RIF1 recruitment to a sequence-defined DSB site was determined quantitatively by ChIP followed by quantitative polymerase chain reaction (qPCR). Cells were crosslinked with 1% (v/v) formaldehyde for 10 min and then glycine was added to a final concentration of 125 mM for 5 min to stop the crosslinking reaction. Cells were lysed in lysis buffer (25 mM HEPES pH 7.9, 300 mM sucrose, 50 mM NaCl, 1 mM EDTA, 3 mM MgCl_2_, and 0.5% Triton X-100) and nuclei were isolated. Nuclear fractions were resuspended in sonication buffer (50 mM HEPES pH 7.9, 140 mM NaCl, 1 mM EDTA, 1% Triton X-100, 0.1% sodium deoxycholate, 1% SDS, 1× protease inhibitor cocktail, and 1× phosphatase inhibitor cocktail (Roche)) for 10 min on ice and sonicated to obtain approximately 200–500-bp chromatin fragments using a Bioruptor (Diagenode). Chromatin fragments were precleared with magnetic Dynabeads protein G (Life Technologies) for 1 h and incubated with pre-bound antibody–Dynabeads protein G overnight at 4 °C. Beads were washed once in low-salt buffer (20 mM Tris, pH 8.1, 2 mM EDTA, 50 mM NaCl, 1% Triton X- 100, and 0.1% SDS), once in high-salt buffer (20 mM Tris, pH 8.1, 2 mM EDTA, 500 mM NaCl, 1% Triton X-100, and 0.1% SDS), once in LiCl buffer (10 mM Tris, pH 8.0, 1 mM EDTA, 0.25 mM LiCl, 1% Nonidet P-40, and 1% deoxycholic acid), and twice in TE buffer (10 mM Tris-HCl, pH 8.0, and 1 mM EDTA). Washed beads were eluted twice with 100 µL of elution buffer (1% SDS and 0.1 M NaHCO_3_) and crosslinks were reversed by overnight incubation at 65 °C in 0.1 mg/mL RNase A, 0.3 M NaCl, and 0.2 mg/mL proteinase K. The DNA samples were purified with Qiaquick PCR columns (Qiagen). qPCR was carried out on an Applied Biosystem 7900 HT Fast instrument using the SYBR Green detection system. The results of the quantitative ChIP assays are the mean with s.e.m. of qPCR reactions from three independent experiments and primers used are listed in Supplementary Tables 4 and 5.

### ER-*Asi*SI resection assay

The percentage of resection adjacent to a specific DSB1 (Chr 1: 89231183) was measured as described^42^ with some modifications. The primer pairs for “DSB1” and “DSB2” are across *Bsr*G1 and *Bam*H1 restriction sites, respectively. Briefly, ER-*Asi*SI U2OS cells were treated with 300 nM of 4-OHT (Sigma) for 3 h to allow the nuclear translocation of *Asi*SI and the induction of DSBs. Cells were collected and genomic DNA was extracted and digested with *Bsr*GI or *Bam*H1 enzymes or mock digested overnight at 37 °C. Digested or mock-digested samples were used as a template for qPCR performed using SYBR Green master mix. Primers used are listed in Supplementary Table 6^42^. For each sample, a ΔCt was calculated by subtracting the Ct value of the mock-digested sample from the Ct value of the digested sample.

### Protein purification

PARP-1, PARP-2, and PARP-3 were purified according to standard procedures^69,70^. BLM was tagged at the N-terminus with GST and at the C-terminus with His_10_ and purified as described for PALB2^70^. MRE11-RAD50-NBS1 was purified according to an established protocol^71^. RPA was purified as described^72^. Human EXO1^30^ or biotinylated EXO1 for single-molecule experiments was purified as described^73^. For recombinant DNA2 protein purification, Sf9 insect cells (1 L at 10^6^ cells/mL) were infected with a GST-DNA2-FLAG baculovirus. At 48 h post-infection, cells were harvested by centrifugation and the pellet was frozen on dry ice. Cells were lysed in Buffer 1 (1× PBS containing 150 mM NaCl, 1 mM EDTA, 0.05% Triton X-100, 1 mM DTT, and protease inhibitors) and homogenized by 20 passes through a Dounce homogenizer (pestle A). The cell lysate was incubated with 1 mM MgCl_2_ and 2.5 U/mL benzonase nuclease at 4 °C for 1 h followed by centrifugation at 93,753 × *g* for 1 h. The soluble cell lysate was incubated with 1 mL of GST-Sepharose beads for 90 min at 4 °C with gentle rotation. The beads were washed twice with buffer 1 followed by incubation with buffer 2 (Buffer 1 with 5 mM ATP, 15 mM MgCl_2_) for 1 h at 4 °C. Sepharose GST beads were washed twice with buffer 1 supplemented with 200 mM NaCl and once with P5 buffer (50 mM NaHPO_4_ pH 7.0, 500 mM NaCl, 10% glycerol, 0.05% Triton-X-100, 5 mM imidazole) followed by cleavage with PreScission protease (60 U/mL, GE Healthcare Life Sciences), overnight at 4 °C in P5 buffer. The supernatant was then collected and completed to 10 mL with Flag-binding buffer (50 mM Tris–HCl pH 7.5, 150 mM NaCl, 1 mM EDTA, 10% glycerol, 0.025% Triton X-100) before incubation with 600 µL of M2 anti-Flag affinity gel (Invitrogen) for 1 h at 4 °C. The beads were washed twice with washing buffer (Flag-binding buffer supplemented with 100 mM NaCl). After two additional washes with Flag Elution buffer (50 mM Tris–HCl pH 7.5, 150 mM NaCl, 0.025% Triton X-100, and 10% glycerol), proteins were eluted twice in one volume of beads with Flag Elution buffer and 500 µg/mL of 3×-Flag peptide for 45 min at 4 °C. Proteins were then dialyzed in the storage buffer (20 mM Tris-HCl, pH 7.4, 200 mM NaCl, 10% glycerol, 1 mM DTT) and stored in aliquots at −80 °C.

### DNA resection assays

Assays were performed with pUC19 DNA linearized with *Kpn*I and then 3′ labeled with [α-^32^P] ATP and terminal deoxytransferase (NEB). For the DNA2 resection machinery, reactions were conducted using 50 nM of substrate in standard buffer (20 mM Na-HEPES pH 7.5, 0.1 mM DTT, 0.05% Triton X-100, 100 µg/mL BSA). Two millimolar ATP and 5 mM MgCl_2_ were added to the reaction buffer immediately before reconstitution of the resection machineries. The reactions were initiated on ice by adding either NAD, PARP-1, PARP-2 or PARP-3 as indicated in the figure and transferred immediately to 37 °C. After 5 min, the order of addition and incubation of the respective protein components were: MRN (10 nM, 5 min), RPA (100 nM, 5 min), BLM (15 nM, 3 min) and DNA2 (15 nM, 45 min). For the EXO1 resection machinery, reactions were conducted using resection buffer (25 mM MOPS pH 7, 60 mM KCl, 1% Tween 20, 2 mM DTT, 5 mM MgCl2, 2 mM ATP) and the same proteins and time of incubation as mentioned above except for EXO1 at a concentration of 10 nM instead of DNA2. Reactions were followed by proteinase K treatment for 30 min at 37 °C. Products were analyzed on a 1% native agarose gel. Gels were dried on DE81 paper (Whatman) and signals were detected by autoradiography. Densitometric analyses were performed using the FLA-5100 phosphorimager (Fujifilm) and quantified using the Image Reader FLA-5000 v1.0 software.

### DNA-binding assays

The DNA-binding reactions (10 μL) contained ^32^P-labeled DNA oligonucleotides (100 nM) and the indicated concentrations of PARP-1 and NAD in resection buffer (20 mM Na-HEPES pH 7.5, 0.1 mM DTT, 0.05% Triton X-100, 100 μg/mL BSA, 5 mM MgCl_2_, 2 mM ATP). Reaction mixtures were incubated at 37 °C for 10 min and then protein–DNA complexes were fixed with 0.2% (v/v) glutaraldehyde for 15 min. The reactions were subjected to electrophoresis on an 8% 1×-TBE-acrylamide gel and ^32^P-labeled DNA was visualized by autoradiography. Sequences of the oligonucleotides are found in Supplementary Table 7.

### Single-molecule imaging and DNA curtains

Single-molecule DNA curtain data were collected at 37 °C using a Nikon Ti-E microscope in a prism-TIRF configuration. Data were collected with a 200-ms exposure through a ×60 water-immersion objective (1.2 NA, Nikon), a 500-nm long-pass (Chroma), and a 638-nm dichroic beam splitter (Chroma) for two-channel detection through two EMCCD cameras (Andor iXon DU897, cooled to −80 °C). Histograms of PARP-1-binding preference on DNA were acquired by fitting a two-dimensional Gaussian to each individual molecule and finding the center of the fit relative to the barrier position using a custom-written FIJI script (available upon request). A Gaussian curve with a constant offset was fit to the largest peak of the histogram using a custom script written in MATLAB (available upon request). The constant offset in the fitting accounts for molecules that bind nonspecifically along the length of the DNA substrate. The center of the fit is reported in the corresponding figure panel.

For DNA curtains, microscope slides with microfabricated chromium barriers were encased in a microfluidic flowcell and passivated with a fluid lipid bilayer. DNA molecules (λ, 48.5 kb) with biotinylated ends were bound to a subset of the lipids via a biotin–streptavidin interaction. The flowcells were attached by nanoports to a syringe pump-controlled microfluidics system.

λ–DNA substrates for DNA curtains were purchased from NEB and annealed with two oligonucleotides: IF7: (5′-[p]AGG TCG CCG CCC[Biotin]- 3′) and LM3: (5′- [p]GGG CGG CGA CCT TTT TTT TTT TTT TTT TTT TTT TTT TTT TTT TTT TTT TTT TTT TTT TTT TTT TTT TTT TTT TTT TTT TTT TTT TTT TTT -3′). This generated a substrate that had a biotin for attaching to the flowcell surface on one side and a 78-nt 3′ overhang on the other side, which was suitable for Exo1 loading. For visualizing the DNA, YoYo-1 was injected into the flowcell with a glucose oxidase/catalase mixture.

To perform PARP-1 labeling, 300 nM mouse anti-6xHis antibody (Clontech) was pre-incubated with 400 nM anti-mouse secondary QDots (Invitrogen, 605 or 705 depending on labeling strategy) in a 5 µL volume for 10 min on ice. PARP-1 or PARP-2 were incubated with the antibody mixture for another 10 min on ice and diluted to a final volume of 200 µL (6.25 nM PARP, 7.5 nM anti-His antibody, 10 nM QDots; final concentrations). PARP-1 or PARP-2 were injected onto the microscope flowcell at 200 µL/min in loading buffer (40 mM Tris-HCl pH 8, 200 µg/mL BSA, 2 mM DTT, 2 mM MgCl_2_). After binding, the flowcell was switched to EXO1 resection buffer (40 nM Tris-HCl pH 8, 60 mM NaCl, 200 µg/mL BSA, 2 mM DTT, 2 mM MgCl2, 1 mM ATP) for subsequent EXO1 loading.

EXO1 loading was performed by pre-incubating 100 nM Streptavidin QDots 605 with 80 nM EXO1-biotin in 10 µL EXO1 loading buffer (40 mM Tris-HCl pH 8, 60 mM NaCl, 200 µg/mL BSA, 2 mM DTT, 2 mM MgCl2, 1 mM ATP) on ice for 10 min. Then the mixture was diluted to a final volume of 200 µL in EXO1 loading buffer plus free biotin (4 nM EXO1, 5 nM QDots) and injected onto DNA curtains at 200 µL/min. To prevent dye-induced changes in protein–DNA interactions, YoYo-1 was omitted in experiments involving PARP-1/EXO1 and PARP-2/EXO1.

### Single-molecule analysis of resection tracks

The indicated cell lines or siRNA transfected cells were incubated with 10 µM BrdU for 48 h. After 48 h, the cells were treated with 5 µM BMN 673 for 1 h before IR (10 Gy and released for 1 h). Cells were harvested and counted, 1 × 10^5^ cells per condition were spun down, and resuspended in the resuspension buffer from the FiberPrep DNA Extraction Kit (Genomic Vision). Agarose plugs and DNA solutions were made according to the FiberPrep DNA extraction Kit. Using the FiberComb (Genomic Vision), the DNA was stretched onto a Combicoverslip. The cover slips were baked for 2 h at 60 °C, then incubated in primary antibody (1:100 anti-BrdU) in 5% PBS–BSA for 2 h at 37 °C. The coverslips were washed with PBS-T 3 times for 3 min with shaking and incubated for 1 h in the appropriate secondary antibody (1:200) conjugated to a fluorophore in a humidified chamber at 37 °C. The slides were washed with PBS-T 3 times for 3 min. If needed, YOYO™-1 Iodide staining (1:1000) was performed for 10 min. The slides were washed with ddH_2_O for 1 min. The DNA is then dehydrated by submerging it for 1 min in 70%, 90%, and 100% ethanol sequentially and visualized on a DMI6000B microscope. Fiber length was evaluated by Image J analysis (version 1.51k).

### Survival assays

Cells were seeded in triplicates into a Corning 3603 black-sided clear bottom 96-well microplate at a density of 2000 cells per well. Once attached, the media was changed to include the desired concentration of BMN 673. One hour after BMN 673 treatment, the plate was irradiated with 7.5 Gy. The plates were incubated for 120 h. The nuclei were stained with Hoechst 33342 (Invitrogen) at 10 μg/mL in media for 30 min at 37 °C. Images of entire wells were acquired at ×4 with a Cytation 5 Cell Imaging Multi-Mode Reader followed by quantification of Hoechst-stained nuclei with the Gen5 Data Analysis Software v3.03 (BioTek Instruments). Cell viability was expressed as the percentage of survival in BMN673-treated cells relative to vehicle (DMSO)-treated cells.

### HR in cellulo reporter assays

Dox-inducible I-*Sce*I/DRGFP cell line (TRI-DR-U2OS) was treated without/with 10 µg/mL Dox and BMN 673 (1 µM) or ABT-888 (5 µM) and incubated for 48 h. HR efficiencies were analyzed by flow cytometry after 48-h incubation. HR efficiency was expressed as the percentage of GFP-positive cells. Samples were analyzed in triplicate. Values are expressed as mean and s.e.m.

### Statistical analyses

All data are representative of three or more independent experiments. Prism ver 6.0 was used to do the statistical analyses.

### Reporting summary

Further information on research design is available in the Nature Research Reporting Summary linked to this article.

## Supplementary information

Supplementary Information

Reporting Summary

Source Data

## Data Availability

The source data and uncropped gel pictures underlying Figs. 1A–G, 2B–D, 3A, B, D, E, 4A–F, 5C, F, H, 6B, D, E, and 7A–E and Supplementary Figs. 1A, B, 2A, B, 3A–G, 4A, B, D, E, 5A–F, 6A–G, 7A–C, 8B–D, and 9B–D are provided as a source data file. Data of this study are available from the authors upon reasonable request.
