# Supplementary material for: Poly(ADP-ribose) polymerase-1 antagonizes DNA resection at double-strand breaks
Source: Nat Commun. 2019 Jul 4;10:2954. doi: 10.1038/s41467-019-10741-9 (PMC6609622; doi:10.1038/s41467-019-10741-9)
Supplement: Supplementary file 1 — Supplementary Information [file 41467_2019_10741_MOESM1_ESM.pdf]

SUPPLEMENTARY INFORMATION

Poly(ADP-ribose) polymerase-1 antagonizes DNA resection at double-strand breaks

Caron, Sharma et al.

Supplementary Table 1. List of antibodies used in this study

REAGENTS	SOURCE	DILUTION USED	IDENTIFIER
**Antibodies**			
PARP-1	Abcam	1:4000 (WB)	ab194586
PARP-1 (C2-10)	Homemade	1:5000 (WB)	This study
PARP-2	Abcam	1:4000 (WB)	ab176330
β-Actin	Sigma	1:10000 (WB)	A5316
RIF1	Bethyl	3μl each sample (CHIP)	A300-569A
53BP1	Novus	3μl each sample (CHIP)	NB100-304
RPA2	Abcam	3μl each sample (CHIP)	ab76420
RPA2	Calbiochem	1:1000 (WB)	NA18
RPA2 S4/S8	Novus	1:1000 (WB)	NBP1-23017
BrdU	GE healthcare	1:750 (IF)	RPN202
Cyclin A	BD Biosciences	1:300 (IF)	611264
RPA2	Abcam	1:2000 (IF)	ab2175
γ-H2AX	Active motif	1:2000 (IF)	39117
BRCA1	Millipore	1:1000 (WB)	07-434
BRCA2	Millipore	1:250 (WB)	OP95
53BP1	Novus	1:2000 (WB)	NB100-304
53BP1	Novus	1:500 (IF)	NB100-304
Histone H3	Abcam	1:10 000 (WB)	Ab1791
GAPDH	Fitzgerald	1:20 000 (WB)	10R-G109A
Alpha-tubulin (DM1A)	Abcam	1:100 000 (WB)	Ab7291
RIF1	Bethyl	1:400 (IF)	A300-569A
CtIP	Active Motif	1:400 (IF)	61141
CtIP	Active Motif	1:3000 (WB)	61141
Geminin	Proteintech	1:300 (IF)	10802-1-AP
Alexa Fluor 488 goat anti-rabbit	Molecular probes	1:800 (IF)	A11008
Alexa Fluor 488 goat anti-mouse	Molecular probes	1:1000 (IF)	A11001

Alexa 568 goat anti-mouse	Molecular probes	1:800 (IF)	A11004
Alexa 568 goat anti-rabbit	Molecular probes	1:800 (IF)	A11011
Ku80	Cell Signalling	1:3000 (WB)	2180S
DNA Ligase IV	Abcam	1:3000 (WB)	ab193353
His tag	Clontech		631212
GFP	Abcam	1:8000 (WB)	Ab290
Alexa488 goat anti-rabbit	Life technology	1:400 (IF)	A11034
Mouse Cy3 goat anti-mouse	Jackson ImmunoResearch	1:400 (IF)	115-165-146
RAD51	Cedarlane Labs	1:1000 (IF)	70-001
QDot 605 streptavidin conjugate	Thermo Fisher Scientific	5 nM (final working concentration)	Q10101MP
QDot 605 anti-mouse conjugate	Thermo Fisher Scientific	10 nM (final working concentration)	Q11001MP
QDot 705 anti-mouse conjugate	Thermo Fisher Scientific	10 nM (final working concentration)	Q11062MP
YOYO™-1 Iodide	Thermo Fisher Scientific	1 nM (final working concentration)	Y3601
Anti-BrdU	GE Healthcare	1:100 (SMART)	RPN202
YOYO™-1 Iodide	Invitrogen	1:1000 (SMART)	Y3601
DNA PKcs(phospho S2056)	Abcam	1:500 (IF)	Ab18192
pADPr (10H)	Tulip Biolabs	1:5000 (WB)	1020
pATR(Thr1989)	Genetex	1:1000 (WB)	GTX128145
Vinculin	Sigma	1:100 000 (WB)	V9131
Phospho-EXO1	Homemade	1:1000 (WB)	Kum Kum Khanna https://doi.org/10.1093/nar/gkp1164

Supplementary Table 2. Reagents or resources used in this study

REAGENT or RESOURCE	SOURCE	IDENTIFIER
Chemicals, Peptides, and Recombinant Proteins		
Proteinase K	Sigma	P2308-100MG
Bromodeoxyuridine (BrdU)	Sigma	B5002
BMN 673	MedChem Express	HY-16106
Veliparib	MedChemExpress	HY-10129
Pre-stained Protein ladder	Frogga Bio	PM008-0500F
RNAiMAX	Invitrogen	13778-075
Oligofectamine	Thermo Fisher Scientific	12252011
Effectene	Qiagen	301425
BSA	Sigma	A7906
Fetal Bovine serum	Gibco	12483-020
Charcoal striped Serum	Sigma	F6765-100ML
Trypsin	Sigma	T4049-500ML
Hygromycin B	invitrogen	10687010
Shield1	TaKaRa	632189
Puromycin	Sigma	P9620-10ML
4-Hydroxytamoxifen (4-OHT)	Sigma	H7904-5MG
BsrG1	NEB	R0575S
BamH1-HF	NEB	R3136S
Bright Green 2x qPCR MasterMix-ROX	abm	MasterMix-R
DMEM high glucose	Fisher scientific	10063542
G418-Geneticin	Gibco	11811-031
Dynabeads Protein G	Invitrogen	10003D
Engraved Combicoverslips	Genomic Vision	COV-002-RUO

[α-32P] ATP	Perkin Elmer	NEG512H250UC
[γ-32P] ATP	Perkin Elmer	BLU002H250UC
NAD	Sigma	N0632
Terminal Transferase	NEB	M0315S
ProLong[R] Gold Antifade Mountant	Invitrogen Life Technology	P-36930
Critical Commercial Assays		
FiberPrep[R] (DNA Extraction Kit)	Genomic Vision	EXTR-001
Click-iT EdU Alexa Fluor[TM] 488 Imaging Kit	Thermo Fisher Scientific	C10337
**Experimental Models: Cell Lines**		
PARP-1 HeLa SilenciX	Tebu-bio	01-00019
Control Hela SilenciX	Tebu-bio	01-00001
U2OS	ATCC	HTB-96
U2OS CRISPR PARP-1(-/-)	This study	N/A
HeLa	ATCC	CCL-2
HEK 293T	ATCC	CRL-11268
HEK 293T CRISPR PARP-1 (-/-)	This study	N/A
ER-AsiSI U2OS	Gaëlle Legube	N/A
TRI-DR-U2OS	Philipp Oberdoerffer	N/A
DLD1 (BRCA2 +/-)	Scott Kern	N/A
DLD1 (BRCA2 -/-)	Scott Kern	N/A
HEK 293	ATCC	CRL-1573
U2OS-RPA2-GFP(stable)	This study	N/A
U2OS-DSB reporter cell line (Fok1 system)	Roger A Greenberg	N/A
Sf9	ThermoFisher	B82501
U2OS-Ku80-GFP(stable)	This study	N/A
MEFs PARP1 +/+	This study	N/A

MEFs PARP1-/-	This study	N/A
**Oligonucleotides**		
siRNA sequences	This paper	Table S3
qPCR primer sequences used in ER-*Asi*SI CHIP experiment	This paper	Table S4
qPCR primer sequences used in U2OS-DSB-reporter locus (FoK1) CHIP experiment.	This paper	Table S5
qPCR primer sequences used in ER-AsiSI resection assay	This paper	Table S6
Sequences of the oligonucleotides used for the bandshift assay	This paper	Table S7
**Recombinant DNA**		
Plasmid: pEGFP-C1-FLAG-Ku80	Addgene	Plasmid#46958
Plasmid: RPA1-GFP	This paper	N/A
Plasmid: RPA2-GFP	This paper	N/A
Plasmid: RPA3-GFP	This paper	N/A
Plasmid : GFP-EXO1	This paper	N/A
Plasmid : mCherry-PCNA	Schonenberger et al.	N/A
**Software and Algorithms**		
Prism	GraphPad	Ver-6
Image J	Image J	V1.51k
GDSC ImageJ (Find Foci plugins)	Herbert et al., 2014	http://www.sussex.ac.uk/gdsc/intranet/microscopy/imagej/gdsc_plugins.
Image Reader FLA-5000	FLA-5100 phosphorimager Software	V1.0
Gen5 Data Analysis Software v3.03	Gen5 Data Analysis Software	v3.03

Supplementary Table 3. List of siRNAs used in the study.

Target protein	siRNA name	Sequence 5′-3′
None	siRNA control	GGG AUA CCU AGA CGU UCU ATT dTdT
None	siRNA control	UUCGAACGUGUCACGUCAAdTdT
PARP-1	siPARP-1	AAG AUA GAG CGU GAA GGC GAA dTdT
PARP-1	siPARP-1-2	AAG AGC GAU GCC UAU UAC UGC dTdT
CtiP	CtiP	GGGAACAGCAGAAAGTCCTTCATGA
Ku80	siKu80	SASI_Hs01_00099411 (NM_021141, Sigma)
DNA Ligase IV	siDNA ligase IV	L-004254-00-0005 (Dharmacon)
53BP1	Si53BP1	GGACUCCAGUGUUGUCAUU dTdT
BRCA1	siBRCA1	CAGCAGUUUAUUACUCACUAA dTdT
BRCA2	siBRCA2	GAAGAAUGCAGGUUUAAUAUU dTdT
KU80	siKU80	GCGAGUAACCAGCUCAUAAUU

Supplementary Table 4: qPCR primer sequences used in ER-*Asi*SI CHIP experiment.

Name	Sequence 5′-3′
chr1_89231183: FW	GATTGGCTATGGGTGTGGAC
chr1_89231183: REV	CATCCTTGCAAACCAGTCCT
chr6_90404906: FW	TGCCGGTCTCCTAGAAGTTG
chr6_90404906: REV	GCGCTTGATTTCCCTGAGT

chr21_21292316:FW	TGGCTGGAACTGCTTTCTTT
chr21_21292316:REV	GGTGAGTGAATGAGCTGCAA

Supplementary Table 5: qPCR primer sequences used in U2OS-DSB-reporter locus (FoK1) CHIP experiment.

Name	Sequence 5′-3′
p1: FW	GGAAGATGTCCCTTGTATCACCAT
p1: Rev	TGGTTGTCAACAGAGTAGAAAGTGAA
p4: FW	CCACCTGACGTCTAAGAAACCAT
p4: REV	GATCCCTCGAGGACGAAAGG

Supplementary Table 6: qPCR primer sequences used in ER-AsiSI resection assay.

Name	Sequence 5′-3′
DSB-335 FW	GAATCGGATGTATGCGACTGATC′
DSB-335 REV	TTCCAAAGTTATTCCAACCCGAT
No DSB FW	ATTGGGTATCTGCGTCTAGTGAGG
No DSB Rev	GACTCAATTACATCCCTGCAGCT
DSB2-364 FW	CCAGCAGTAAAGGGGAGACAGA
DSB2-364 REV	CTGTTCAATCGTCTGCCCTTC

Across DSB1 Primer FW	GATGTGGCCAGGGATTGG
Across DSB1 Primer REV	CACTCAAGCCCAACCCGT

Supplementary Table 7: Sequences of the oligonucleotides used for the EMSA assays.

Name	Sequence 5′-3′
JYM696	GGGCGAATTGGGCCCGACGTCGCATGCTCCTCTAGACTCGAGGAA TTCGGTACCCCGGGTTCGAAATCGATAAGCTTACAGTCTCCATTTAA AGGACAAG
JYM698	CTTGTCCTTTAAATGGAGACTGTAAGCTTATCGATTTCGAACCCGGG GTACCGAATTCCTCGAGTCTAGAGGAGCATGCGACGTCGGGCCCAA TTCGCCC

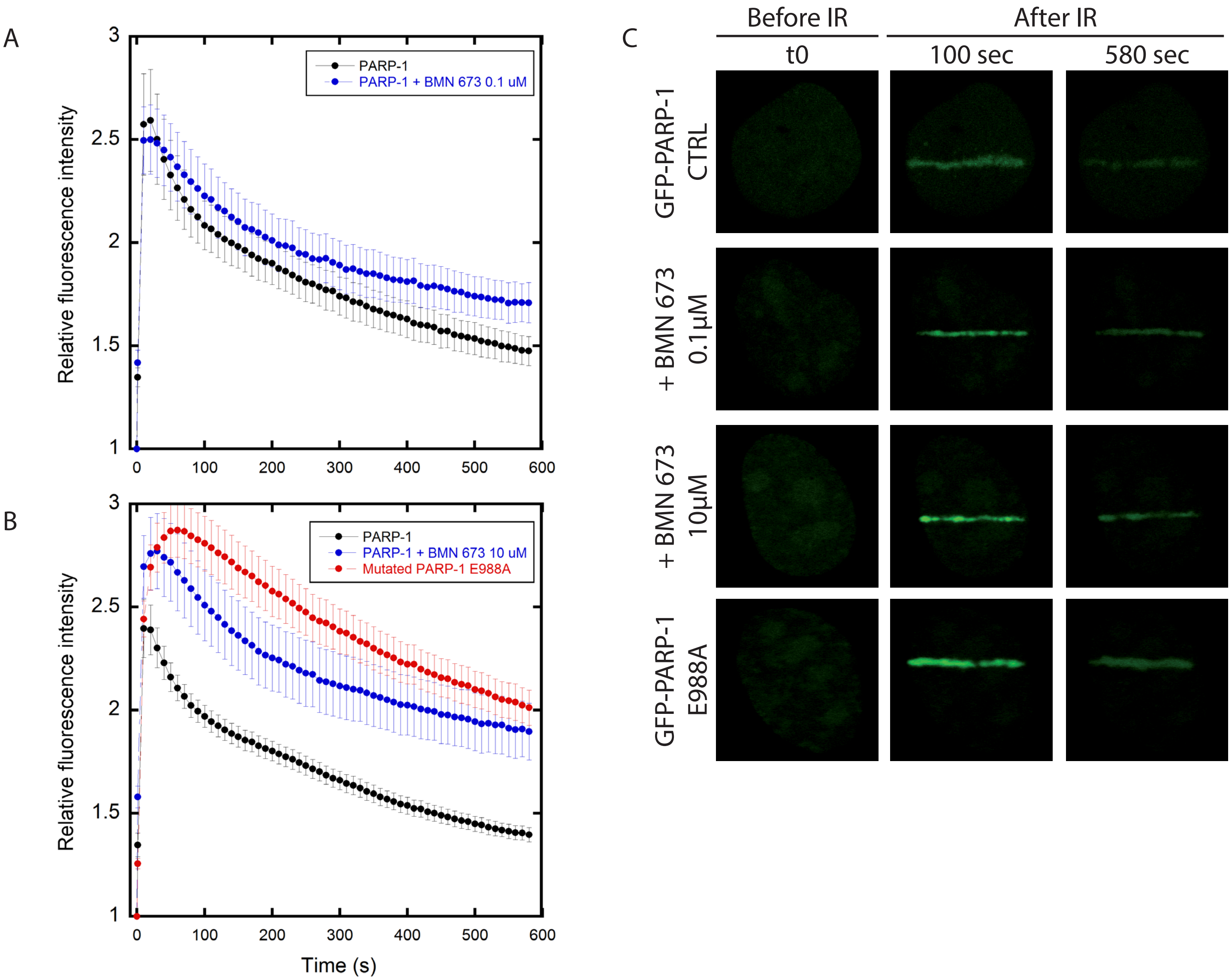

Supplementary Figure 1. Recruitment of PARP-1 at laser-induced DNA breaks. The recruitment dynamics of PARP-1 under normal conditions was compared with the dynamics observed following PARP inhibition. The recruitment kinetics was obtained by plotting the relative fluorescence intensity of GFP-PARP-1 at the damage site as a function of time. (A) Accumulation of PARP-1 in normal conditions or in presence of 0.1µM BMN 673. (B) Accumulation of PARP-1 in normal conditions, in presence of 10µM BMN 673, and of polymerization mutant (E988A). (C) Representative images of local accumulation of GFP-PARP-1 at laser-induced DNA damage sites. Experiments were repeated three times and the average of at least 8 cells from a representative experiment was used to generate the graphs. Two-way analysis of variance followed by Tukey pairwise comparisons shows no significant differences between PARP-1 and PARP-1 + BMN 673 0.1µM but significant differences in time between PARP-1 and PARP-1 + BMN 673 10µM ($P < 0.02$) and between PARP-1 and mutated PARP-1 E988A ($P < 0.01$) (from 20 to 580 seconds). Data show the mean ± s.d. (Tukey test).

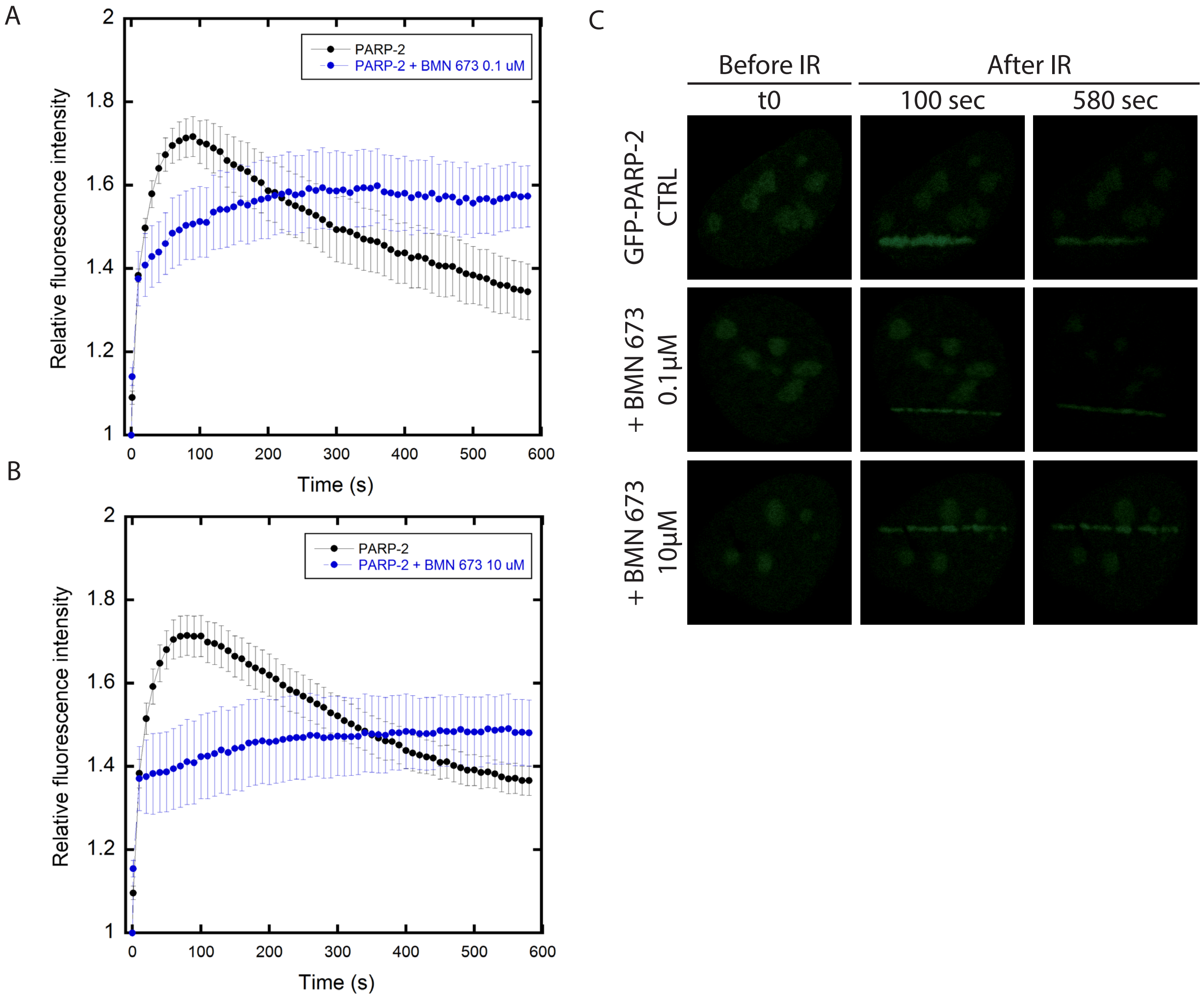

Supplementary Figure 2. Recruitment of PARP-2 at laser induced DNA breaks. The recruitment dynamics of PARP-2 under normal conditions was compared with the dynamics observed following PARP inhibition. The recruitment kinetics was obtained by plotting the relative fluorescence intensity of GFP-PARP-2 at the damage site as a function of time. Accumulation of PARP-2 in normal conditions or in presence of (A) 0.1µM BMN 673 and (B) 10µM BMN 673. (C) Representative images of local accumulation of GFP-PARP-2 at laser-induced DNA damage sites. Experiments were repeated three times and the average of at least 8 cells from a representative experiment was used to generate the graphs. Error bars represent the SEM. By two-way ANOVA, we observed a significant difference between PARP-2 and PARP-2 + BMN 673 0.1µM ($P < 0.002$) and between PARP-2 and PARP-2 + BMN 673 10µM ($P < 0.001$) over time. Data show the mean ± s.d. (Tukey test).

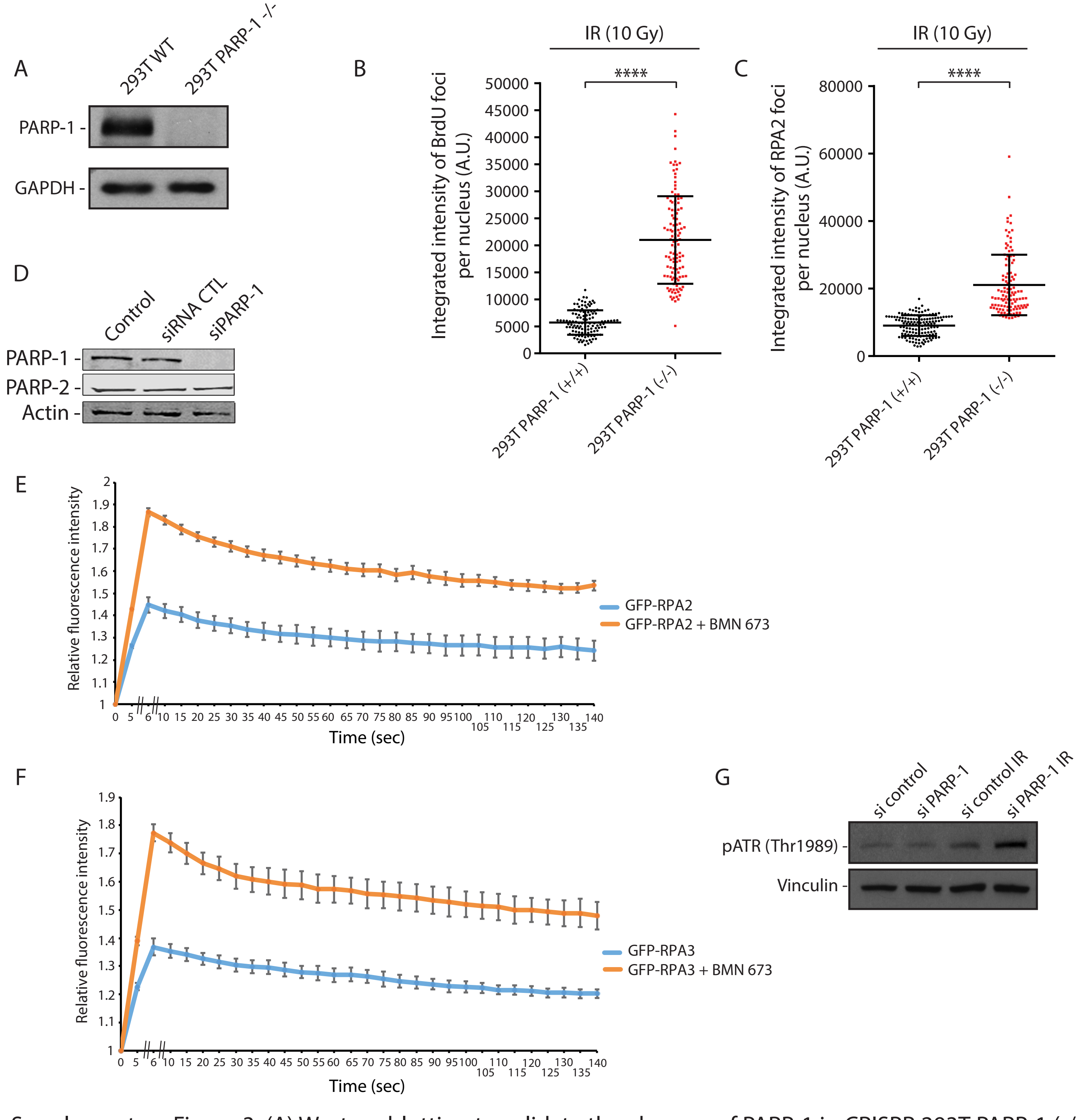

Supplementary Figure 3. (A) Western blotting to validate the absence of PARP-1 in CRISPR 293T PARP-1 (-/-) compared to the 293T PARP-1 (+/+) control. CRISPR 293T PARP-1 (+/+) or 293T PARP-1 (-/-) cells were subjected to immunofluorescence against BrdU (B) or RPA2 (C) after irradiation (10Gy, 3 hrs release). Data in panels B-C show the mean ± s.d. **** $p \leq 0.0001$, (Mann-Whitney U-test). (D) PARP-1 knockdown efficiency monitored by western blotting. The recruitment of GFP-RPA2 (E) or GFP-RPA3 (F) is accelerated by PARP inhibition at laser-induced DNA breaks. HEK293 cells were co-transfected with mCherry-PCNA and RPA subunit 2 or 3. Cells in S-phase, co-expressing mCherry and GFP, were micro-irradiated using 750-nm two-photon laser beam, and the accumulation of RPA at the sites of DNA damage was monitored on a Zeiss LSM 510 NLO laser scanning confocal microscope. The peak of intensity was at 6 seconds. Data show the mean ± s.e.m. (G) ATR is over-activated in the PARP-1 knockdown cells (after 5Gy irradiation and 90 min release).

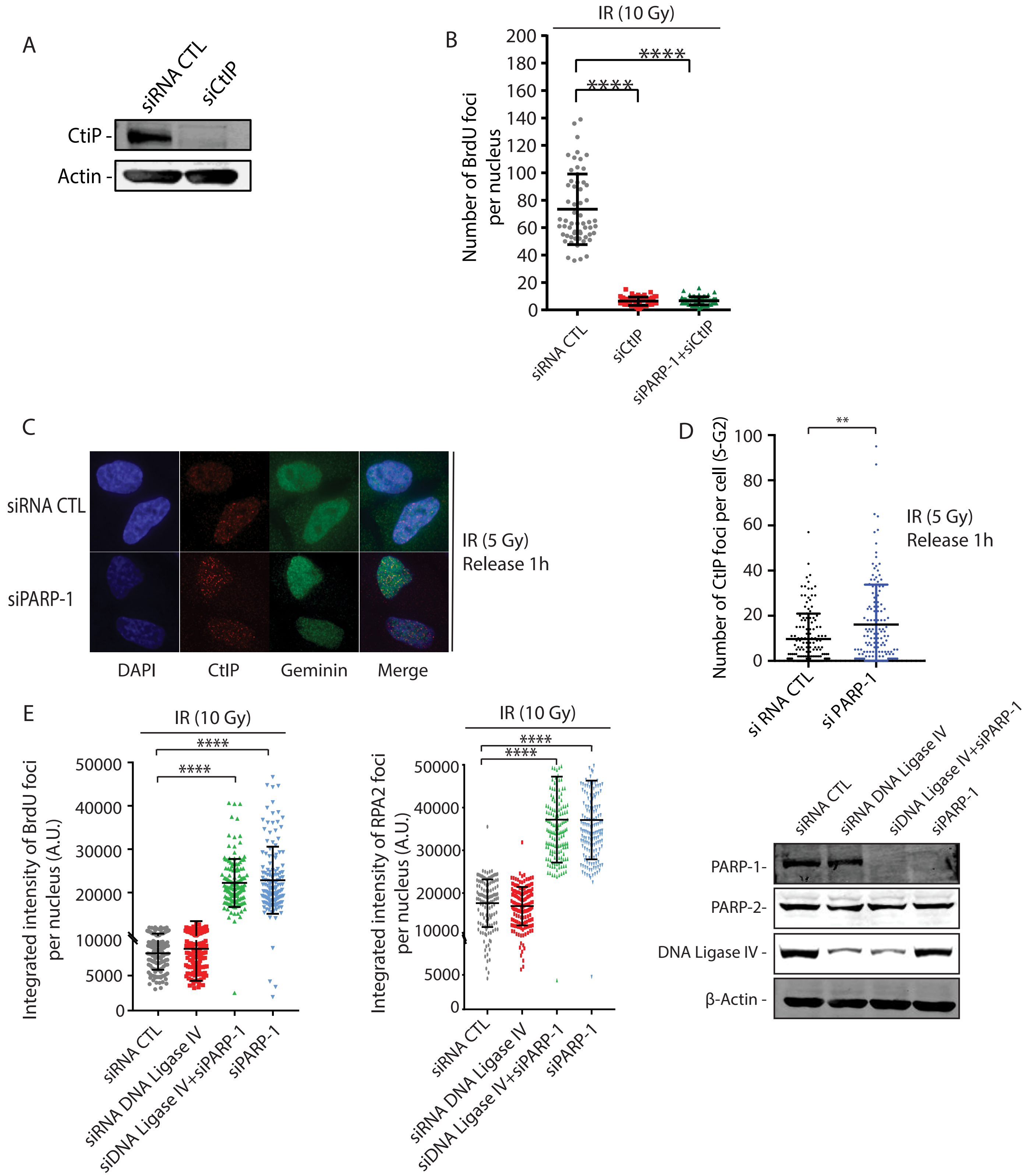

Supplementary Figure 4. (A) Western blotting to monitor CtIP knockdown efficiency. (B) Quantification of the number of BrdU foci in the indicated knockdowns in HeLa SilenciX following irradiation (10 Gy 3hr release). Data show the mean ± s.d. **** $p \leq 0.0001$, (Mann-Whitney U-test).
(C) CtIP foci formation is increased in PARP-1 knockdown cells. (D) Quantification of the results. Data show the mean ± s.d. ** $p \leq 0.01$, (Mann-Whitney U-test).
(E) The indicated knockdown in HeLa SilenciX control or PARP-1 HeLa SilenciX cells were subjected to immunofluorescence against BrdU (left panel) or RPA2 (middle panel) following irradiation (10 Gy, 3 hr release). (Right). Data show the mean ± s.d. **** $p \leq 0.0001$ (Mann-Whitney U-test). Western blotting to validate siRNA knockdowns of DNA ligase IV or/and PARP-1.

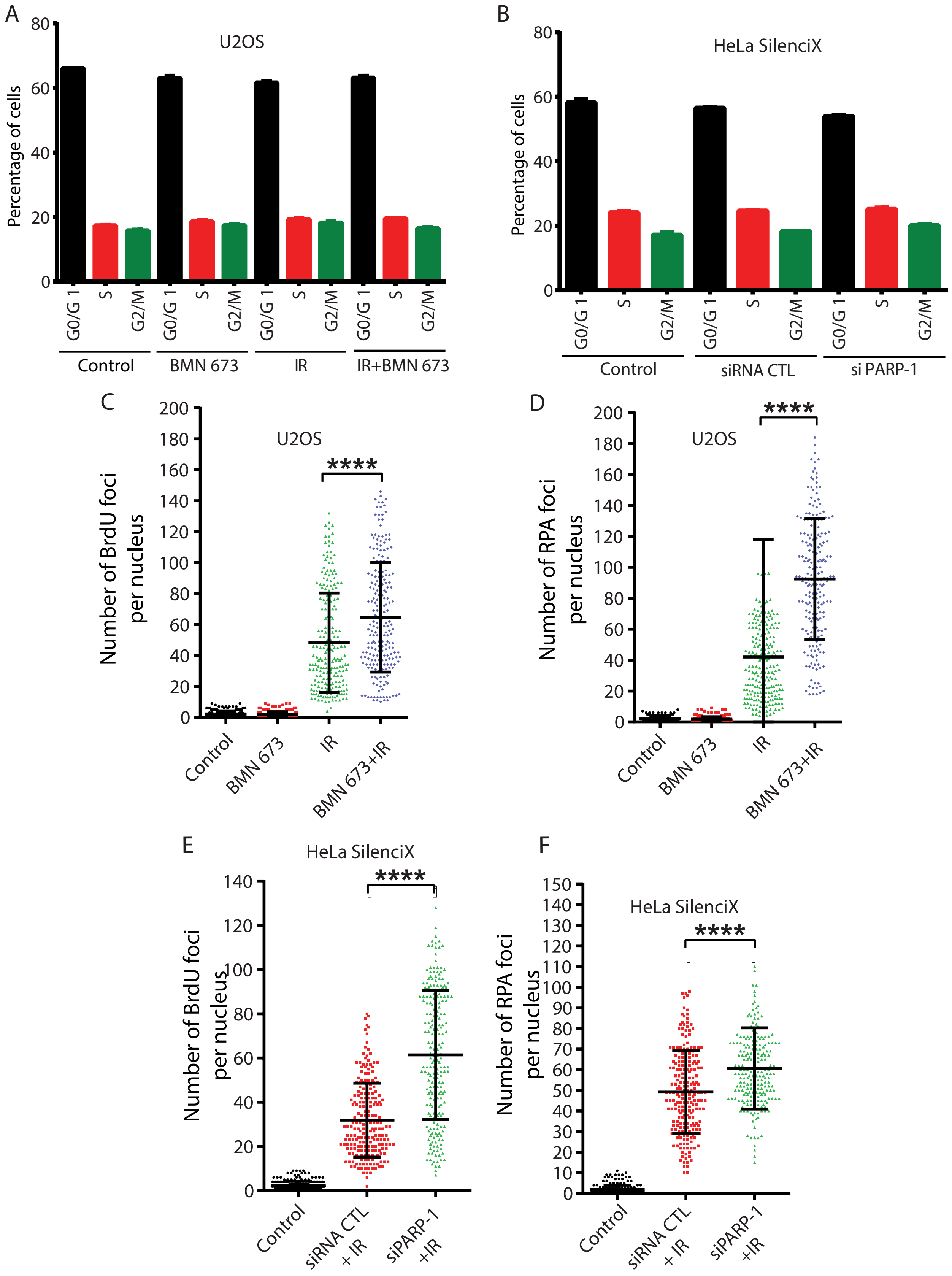

Supplementary Figure 5. (A-B) FACS analysis to monitor the distribution of the indicated cells in each cell cycle phase for the BrdU/RPA analysis (3 hours release from the indicated treatments). Data show the mean ± s.e.m.
U2OS cells either mock treated, treated with BMN 673, irradiated (10 Gy), or irradiated (10 Gy) in combination with BMN 673, were subjected to immunofluorescence against BrdU (C) or RPA2 foci formation (D). The graph represent the number of BrdU/RPA focus per nucleus. HeLa SilenciX cells underexpressing PARP-1 by siRNA-mediated gene knockdown were subjected to immunofluorescence against BrdU (E) or RPA2 foci formation (F).
The graph represent the number of BrdU/RPA focus per nucleus. Data in panels C-F show the mean ± s.d.
**** p ≤ 0.0001, (Mann-Whitney, U-test).

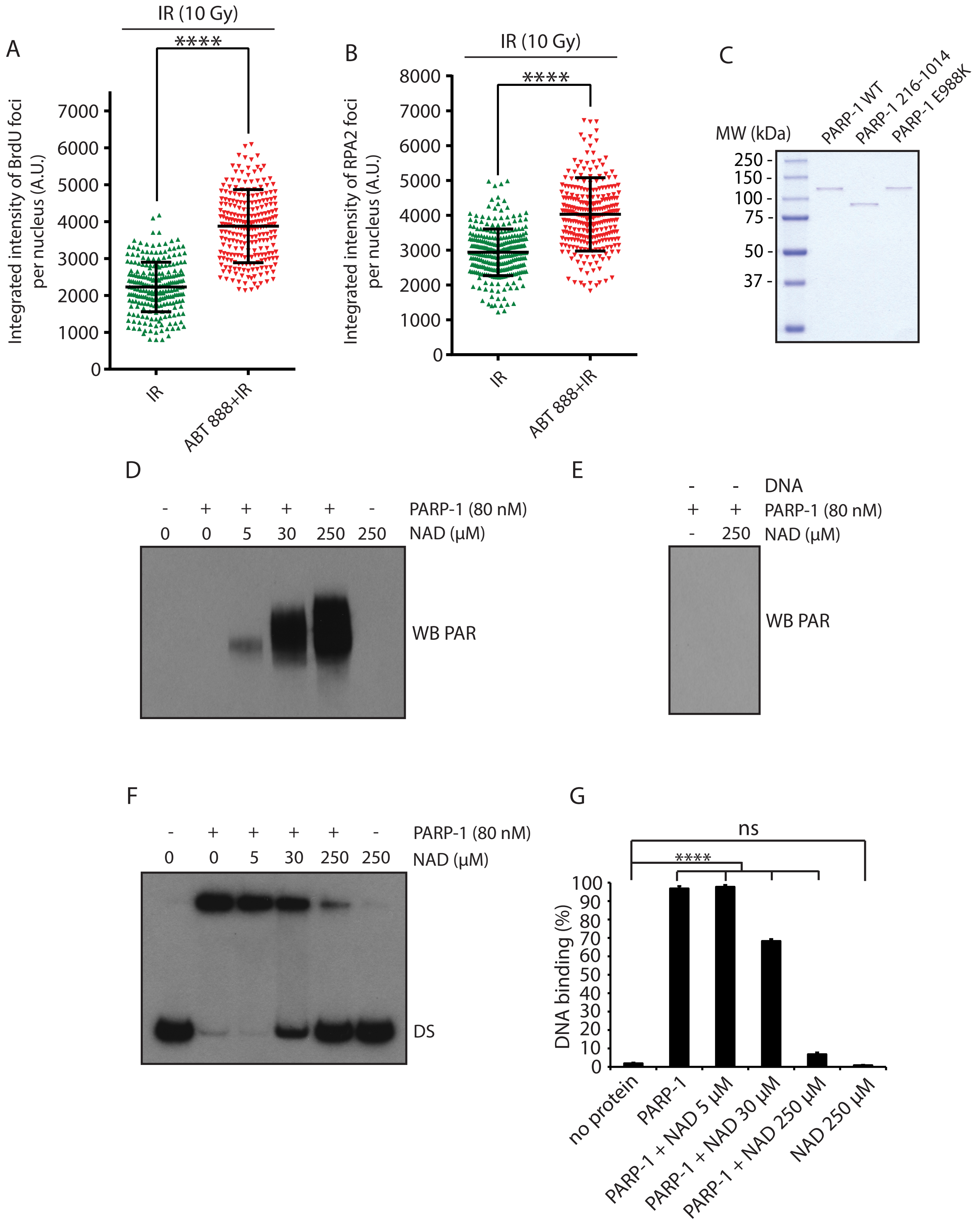

Supplementary Figure 6. U2OS cells were either irradiated (10 Gy), or irradiated (10 Gy) in combination with Veliparib (5 μM), and subjected to immunofluorescence against BrdU (A) or RPA2 foci formation (B). The graph represents the intensity of BrdU/RPA focus per nucleus after 3 hrs recovery. Data in A-B show the mean ± s.d. ****p<0.0001, (Mann-Whitney U-test). (C) SDS-PAGE of purified PARP-1 WT, PARP-1 DNA binding mutant 216-1014, and PARP-1 E988K. (D) PARP-1 was incubated in the resection buffer in the absence or presence of different concentrations of NAD and with 100 nM of dsDNA for 10 minutes at 37°C. PARP-1 activation was then visualized by western blotting using a pADPr antibody. (E) The same reactions were performed as mentioned in (D) without DNA. (F) DNA binding activity of PARP-1 to the dsDNA substrate in the presence or absence of different concentrations of NAD. (G) DNA binding quantifications. Data show the mean ± s.d. ****p<0.0001, (Ordinary one-way Anova).

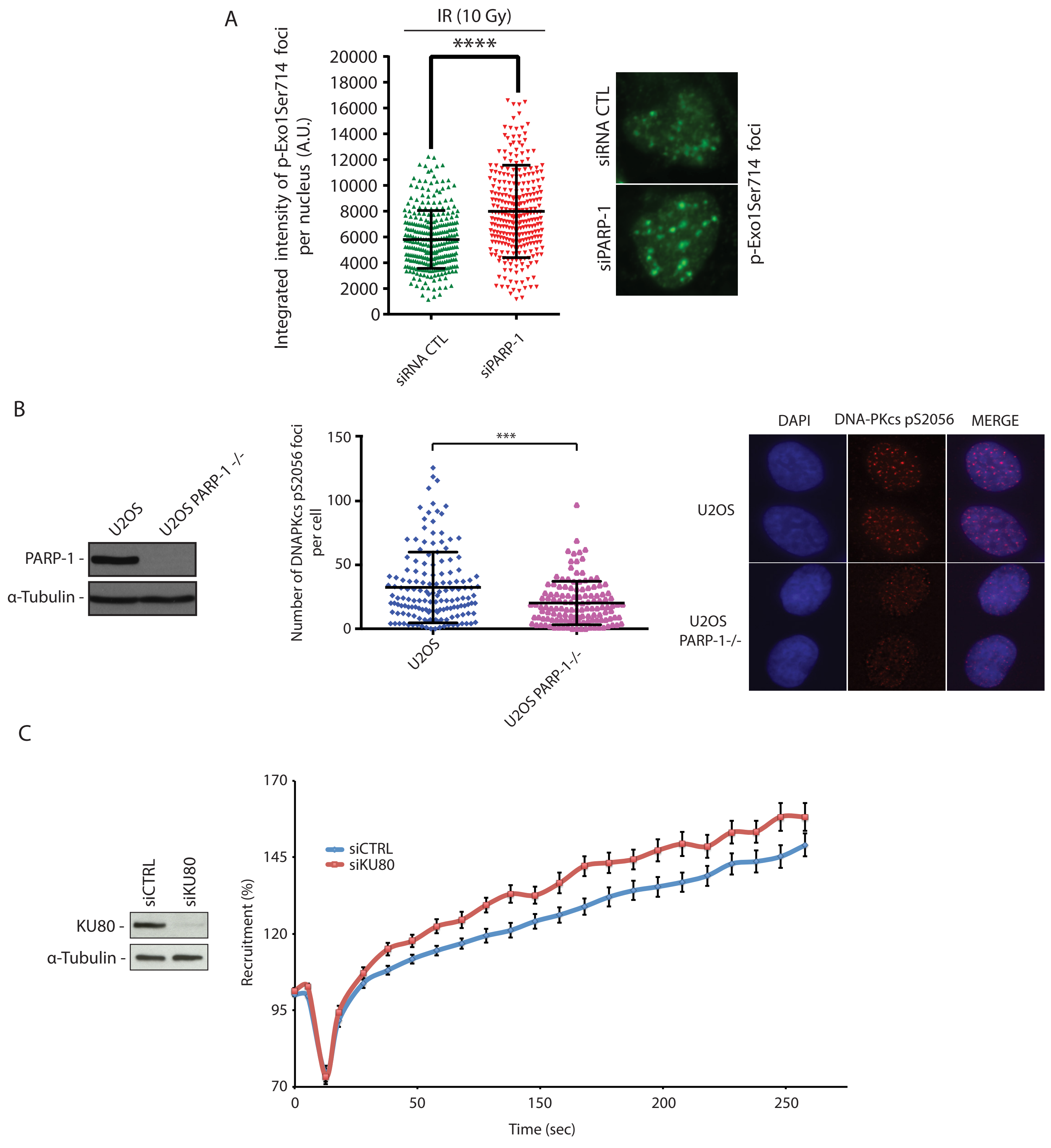

Supplementary Figure 7. (A) Quantification of EXO1 phospho-Ser 714 foci in U2OS cells knockdown for PARP-1 after irradiation (10 Gy, 3 hours release). A representative immunofluorescence picture is shown of the right. The graph represents the integrated intensity of EXO1phospho-Ser 714 foci per nucleus. Data show the mean ± s.d. **** p ≤ 0.0001, (Mann-Whitney U-test). (B) Quantification of DNA-PKcs pS2056 foci in U2OS cells knockout for PARP-1 (left) after irradiation (5 Gy, 1 hour release). Data show the mean ± s.d. *** p ≤ 0.001, (Mann-Whitney U-test). A representative immunofluorescence picture is shown (right).
(C) Left. Western blotting to monitor the knockdown efficiency of Ku80 in HeLa cells. Right. Recruitment of GFP-EXO1 in Ku80 knockdown cells. Data show the mean ± s.e.m.

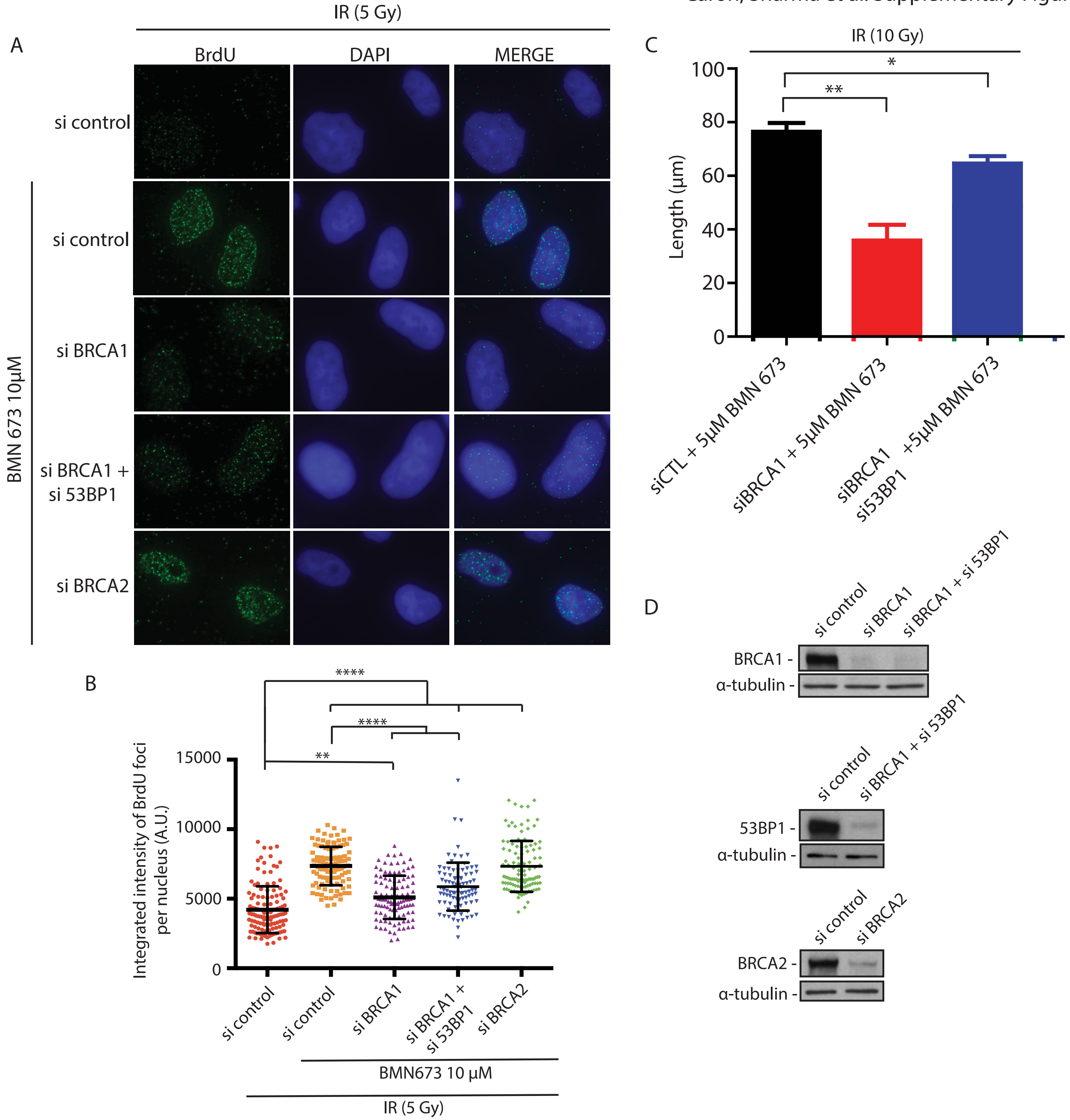

Supplementary Figure 8. (A) In contrast to siRNA inhibition of BRCA2, BRCA1 knockdown decreases single-strand DNA accumulation after irradiation as detected by BrdU staining following irradiation (5 Gy) and BMN 673 treatment.

(B) Quantification of the results by integrated intensity of BrdU foci per nucleus. Data show the mean ± s.d. ** $p \leq 0.01$, **** $p \leq 0.0001$, (Kruskal-Wallis).

(C) SMART assay. HeLa cells were transfected with the indicated siRNAs followed by irradiation (10 Gy) and treatment with BMN 673. 200 fibers were counted in triplicate. Data show the mean ± s.e.m. * $p \leq 0.05$; ** $p \leq 0.01$, (Mann-Whitney).

(D) Western blotting to monitor the knockdown efficiency of BRCA1, 53BP1, BRCA2 knockdown.

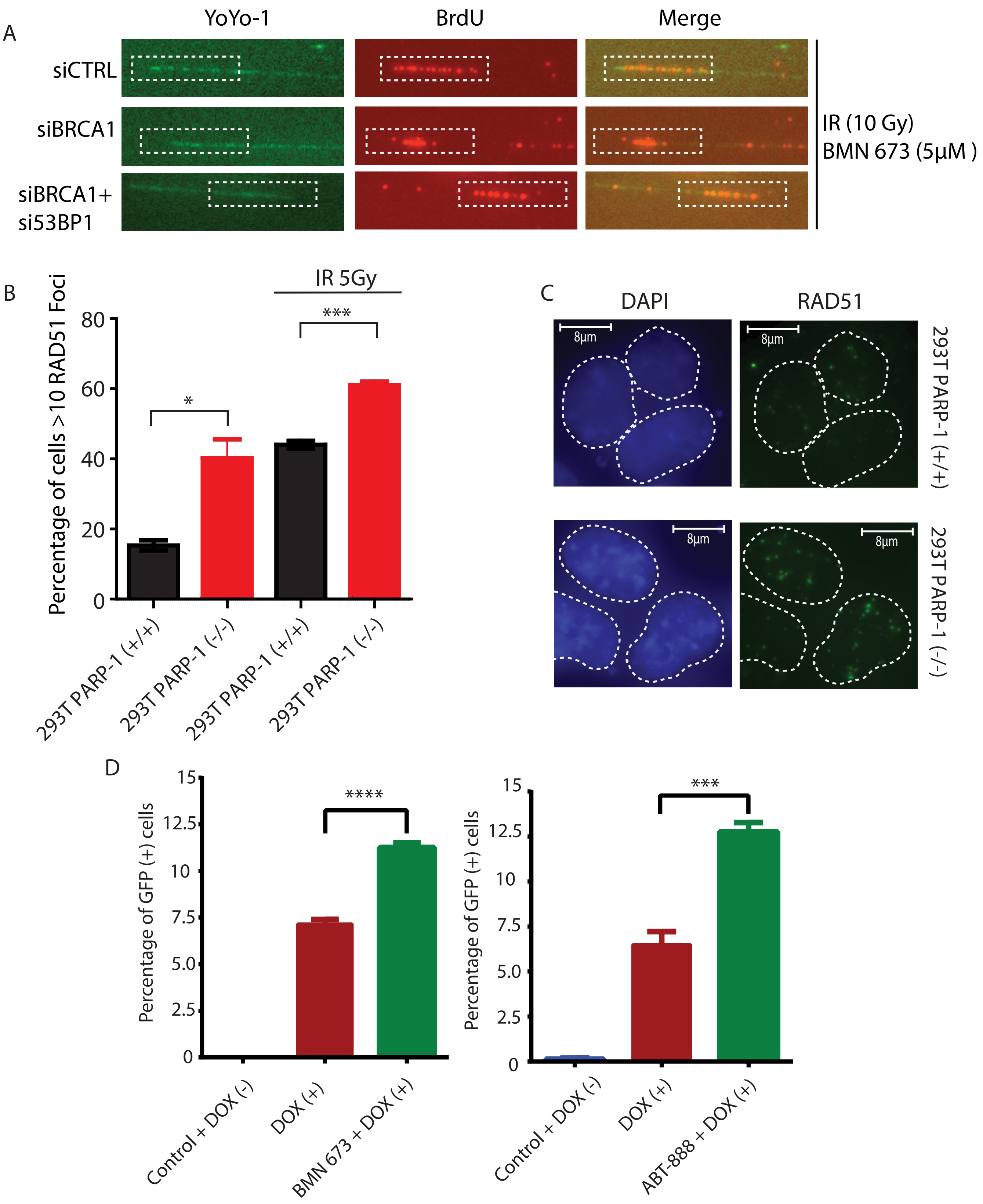

Supplementary Figure 9. (A) Representative pictures of SMART assay samples. DNA tracks were stained with YoYo-1 and an anti-BrdU. The merge picture represent the overlay between the green and red channels. The BrdU signal could not be detected without irradiation.
(B) RAD51 foci formation in CRISPR HEK293T cells proficient and deficient for PARP-1 without or with 5 Gy irradiation. The percentage of cells with more than 10 RAD51 foci were scored. (C) Typical example of RAD51 foci in both CRISPR HEK293T PARP-1 (+/+) and CRISPR HEK293T PARP-1 (-/-) after IR (5 Gy). (D) In cellulo homologous recombination is increased in BMN 673 (left) or ABT-888 (right) treated cells. Data in panels B and D show the mean ± s.e.m. * $p \leq 0.05$, *** $p \leq 0.001$, **** $p \leq 0.0001$ (Mann-Whitney, U-test).
