# Supplementary material for: Poly(ADP-ribose) polymerase-1 antagonizes DNA resection at double-strand breaks
Source: Nat Commun. 2019 Jul 4;10:2954. doi: 10.1038/s41467-019-10741-9 (PMC6609622; doi:10.1038/s41467-019-10741-9)
Supplement: Supplementary file 2 — Reporting Summary [file 41467_2019_10741_MOESM2_ESM.pdf]

nature research

Corresponding author(s): Masson/NCOMMS-18-29469

Reporting Summary

Nature Research wishes to improve the reproducibility of the work that we publish. This form provides structure for consistency and transparency in reporting. For further information on Nature Research policies, see Authors & Referees and the Editorial Policy Checklist.

Statistical parameters

When statistical analyses are reported, confirm that the following items are present in the relevant location (e.g. figure legend, table legend, main text, or Methods section).

n/a	Confirmed	
☐	☒	The exact sample size (*n*) for each experimental group/condition, given as a discrete number and unit of measurement
☐	☒	An indication of whether measurements were taken from distinct samples or whether the same sample was measured repeatedly
☐	☒	The statistical test(s) used AND whether they are one- or two-sided<br>*Only common tests should be described solely by name; describe more complex techniques in the Methods section.*
☒	☐	A description of all covariates tested
☒	☐	A description of any assumptions or corrections, such as tests of normality and adjustment for multiple comparisons
☒	☐	A full description of the statistics including central tendency (e.g. means) or other basic estimates (e.g. regression coefficient) AND variation (e.g. standard deviation) or associated estimates of uncertainty (e.g. confidence intervals)
☐	☒	For null hypothesis testing, the test statistic (e.g. *F*, *t*, *r*) with confidence intervals, effect sizes, degrees of freedom and *P* value noted<br>*Give P values as exact values whenever suitable.*
☒	☐	For Bayesian analysis, information on the choice of priors and Markov chain Monte Carlo settings
☒	☐	For hierarchical and complex designs, identification of the appropriate level for tests and full reporting of outcomes
☒	☐	Estimates of effect sizes (e.g. Cohen's *d*, Pearson's *r*), indicating how they were calculated
☒	☐	Clearly defined error bars<br>*State explicitly what error bars represent (e.g. SD, SE, CI)*

Our web collection on statistics for biologists may be useful.

Software and code

Policy information about availability of computer code

Data collection	N/A
Data analysis	All data is representative of three or more independent experiments. For immunofluorescence studies, at least 100 nuclei were scored by each individual experiment. Prism ver 6.0 was used to do the statistical analyses (Ordinary one-Way ANOVA was used for statistical analysis of biochemical experiments and other experiments were analysed by two-tailed Student's t-test (Mann-Whitney test U-test)), unless mentioned otherwise. The integrated intensity of individual BrdU foci and RPA2 foci were quantified by using GDSC ImageJ Find Foci plugins. Survival assays and nuclei counting were performed using the Gen5 Data Analysis Software v3.03.

For manuscripts utilizing custom algorithms or software that are central to the research but not yet described in published literature, software must be made available to editors/reviewers upon request. We strongly encourage code deposition in a community repository (e.g. GitHub). See the Nature Research guidelines for submitting code & software for further information.

Data

Policy information about availability of data

All manuscripts must include a data availability statement. This statement should provide the following information, where applicable:

- Accession codes, unique identifiers, or web links for publicly available datasets
- A list of figures that have associated raw data
- A description of any restrictions on data availability

The source data and uncropped gel pictures are provided as a source data file.

Field-specific reporting

Please select the best fit for your research. If you are not sure, read the appropriate sections before making your selection.

☒ Life sciences ☐ Behavioural & social sciences ☐ Ecological, evolutionary & environmental sciences

For a reference copy of the document with all sections, see nature.com/authors/policies/ReportingSummary-flat.pdf

Life sciences study design

All studies must disclose on these points even when the disclosure is negative.

Sample size	Not applicable
Data exclusions	Not applicable
Replication	Not applicable
Randomization	Not applicable
Blinding	Not applicable

Reporting for specific materials, systems and methods

Materials & experimental systems

n/a	Involved in the study	
☒	☐	Unique biological materials
☐	☒	Antibodies
☐	☒	Eukaryotic cell lines
☒	☐	Palaeontology
☒	☐	Animals and other organisms
☒	☐	Human research participants

Methods

n/a	Involved in the study	
☒	☐	ChIP-seq
☒	☐	Flow cytometry
☒	☐	MRI-based neuroimaging

Antibodies

Antibodies used	Antibodies used are listed in Supplemental data with the source and identifier (see table of antibodies)
Validation	Antibodies are validate with Western blot or IF with tagged ectopic expression as positive control and siRNA knock down as negative control. For gH2AX, BrdU, and RPA32 Phosphor-S4/S8, cell with and without irradiation was used as positive and negative control.

Eukaryotic cell lines

Policy information about cell lines

Cell line source(s)	All cell lines are listed in Supplemental data with the source and identifier.

Authentication	All cell lines were authenticated by STR profiling at DFCI Molecular Diagnostics Laboratory
Mycoplasma contamination	cell line are all test for mycoplasma with PCR based mycoplasma detection method and only mycoplasma negative cell line were used in study. We test cell line in culture for mycoplasma monthly (e-myco mycoplasm PCR detection kit (25235 LiLif).
Commonly misidentified lines (See ICLAC register)	no commonly misidentified cell lines were used.
